# Classical and Novel TSPO Ligands for the Mitochondrial TSPO Can Modulate Nuclear Gene Expression: Implications for Mitochondrial Retrograde Signaling

**DOI:** 10.3390/ijms18040786

**Published:** 2017-04-07

**Authors:** Nasra Yasin, Leo Veenman, Sukhdev Singh, Maya Azrad, Julia Bode, Alex Vainshtein, Beatriz Caballero, Ilan Marek, Moshe Gavish

**Affiliations:** 1 The Ruth and Bruce Rappaport Faculty of Medicine, Department of Neuroscience, Technion—Israel Institute of Technology, Haifa 32525433, Israel; nasra19@campus.technion.ac.il (N.Y.); mayabz@gmail.com (M.A.); alexanderv21184@gmail.com (A.V.); bea1979c@hotmail.com (B.C.); 2 Faculty of Chemistry, Department of Organic Chemistry, Technion—Israel Institute of Technology, Haifa 3200003, Israel; sukhdev.giri@gmail.com (S.S.); chilanm@tx.technion.ac.il (I.M.); 3 Schaller Research Group at the University of Heidelberg and the German Cancer Research Center (DKFZ), Im Neuenheimer Feld 581, Heidelberg 69120, Germany; j.bode@dkfz-heidelberg.de

**Keywords:** modulation of nuclear gene expression, mitochondrial 18 kDa translocator protein (TSPO), TSPO ligand, PK 11195, 2-Cl-MGV-1, retrograde mitochondrial-nuclear signaling pathway, microscopy, mitochondria, cell nucleus

## Abstract

It is known that knockdown of the mitochondrial 18 kDa translocator protein (TSPO) as well as TSPO ligands modulate various functions, including functions related to cancer. To study the ability of TSPO to regulate gene expression regarding such functions, we applied microarray analysis of gene expression to U118MG glioblastoma cells. Within 15 min, the classical TSPO ligand PK 11195 induced changes in expression of immediate early genes and transcription factors. These changes also included gene products that are part of the canonical pathway serving to modulate general gene expression. These changes are in accord with real-time, reverse transcriptase (RT) PCR. At the time points of 15, 30, 45, and 60 min, as well as 3 and 24 h of PK 11195 exposure, the functions associated with the changes in gene expression in these glioblastoma cells covered well known TSPO functions. These functions included cell viability, proliferation, differentiation, adhesion, migration, tumorigenesis, and angiogenesis. This was corroborated microscopically for cell migration, cell accumulation, adhesion, and neuronal differentiation. Changes in gene expression at 24 h of PK 11195 exposure were related to downregulation of tumorigenesis and upregulation of programmed cell death. In the vehicle treated as well as PK 11195 exposed cell cultures, our triple labeling showed intense TSPO labeling in the mitochondria but no TSPO signal in the cell nuclei. Thus, mitochondrial TSPO appears to be part of the mitochondria-to-nucleus signaling pathway for modulation of nuclear gene expression. The novel TSPO ligand 2-Cl-MGV-1 appeared to be very specific regarding modulation of gene expression of immediate early genes and transcription factors.

## 1. Introduction

Various studies have shown that the 18 kDa translocator protein (TSPO) is involved in numerous functions, including glioblastoma tumorigenicity in its various aspects, e.g., cell proliferation, cell viability, etc. [1,2,3,4,5]. A previous common name for TSPO was peripheral-type benzodiazepine receptor (PBR) [6,7,8]. TSPO is primarily located in the outer mitochondrial membrane [9,10,11]. Thus, TSPO is a mitochondrial protein that interacts with ligands to modulate various molecular biological mechanisms. In 2006, the name translocator protein, and its acronym (TSPO), was generally adopted to reflect TSPO’s participation in transport of molecules over the outer mitochondrial membrane [7,8,12]. Import into mitochondria by the TSPO includes cholesterol, protoporphyrin IX, and other tetrapyrroles [7,12,13]. Release from the mitochondria regulated by TSPO includes Ca^2+^, ATP, and cytochrome c [5,14,15,16]. TSPO is involved in life essential functions, such as respiration, photosynthesis, tetrapyrrole metabolism, and programmed cell death [6,8,17,18]. Extensive studies have shown that TSPO is involved in apoptosis, gene expression, reactive oxygen species (ROS) generation, ATP production, regulation of the mitochondrial membrane potential (ΔΨm), heme synthesis, mitochondrial cholesterol transport, neurosteroid synthesis, glutamate metabolism, cell proliferation, cell adhesion, cell migration, and cell differentiation [3,5,7,12,19,20]. In animals and humans, TSPO shows essential roles in inflammatory, immune, and stress responses, as well as in several neuropathological disorders, including neurodegeneration and brain cancer [4,7,12,21]. Based on this background, we postulated that regulation of nuclear gene expression by the TSPO can go a long way to explain TSPO’s numerous functional effects [18,22].

It has been shown before that regulation of the TSPO by siRNA or application of TSPO ligands can affect gene expression in human cells in culture and bacteria [19,20,21,22,23,24,25,26]. The question we address with this study is whether TSPO is potentially directly involved in mitochondrial capability to regulate nuclear gene expression. First of all, the question became prominent how TSPO can participate in so many functions as mentioned above [1,2,3,13]. We hypothesized that the potential specific capability of TSPO to regulate gene expression may be the basis of TSPO’s general capability to regulate numerous functions [13,18,20,22]. The other angle is that it is well known that mitochondria are able to regulate nuclear gene expression. This phenomenon is called the “retrograde mitochondrial-nuclear pathway for regulation of nuclear gene expression” [27,28,29,30,31]. It is primarily considered as a cellular response to stressors, as the second leg of a nuclear-mitochondrial-nuclear loop. For brevity, from here on we will refer to it as mitochondria-to-nucleus signaling.

Regarding this pathway, mitochondrial ROS generation and loss of Δψm have been reported to take part in mitochondria-to-nucleus signaling [27,32]. This leads to changes in levels of ATP and NADH and the release of Ca^2+^, which activates calcium-sensitive proteins, such as calmodulin and calcineurin. Further downstream this leads to the activation of immediate early genes and transcription factors [28,29,33,34]. This outline of the mitochondria-to-nucleus pathway mirrors well reported observations regarding TSPO’s role in mitochondrial functions and mechanisms. It has been demonstrated that TSPO regulates mitochondrial ROS generation, Δψm transitions, and ATP production [10,11,35,36]. This was originally studied in TSPO’s function of initiating programmed cell death [3,5,37]. Furthermore, the TSPO ligand PK 11195 induces mitochondrial permeability transition pore opening, thus allowing release of Ca^2+^ from the mitochondria into the cytosol [14,38,39]. It has also been suggested that TSPO can regulate NADPH oxidase (NOX) activity [40,41,42,43]. Thus, by regulating ROS generation, Δψm transitions, ATP production, Ca^2+^ release, and NADH levels, TSPO may be a key mitochondrial protein providing mitochondria the capability to regulate gene expression in the cell nucleus.

Therefore, we set out to investigate the effects of the classical TSPO ligand PK 11195 on gene expression in a time course related fashion. We studied this in cell culture of the U118MG glioblastoma cell line, as TSPO functions including gene expression have been studied by us for several years in this cell line (e.g., [5,19,20,24,37,44]). Thereby, we would be able to place the new data in a well-established context. The assumption was that the earlier the changes in gene expression occur, the more likely it is that TSPO takes part in direct control of gene expression, as opposed to secondary effects. Furthermore, we wanted to see whether genes related to transcription, post transcription, translation, and post translation would be affected. When changes in gene expression occur before changes in physiological functions, this would indicate that these changes in gene expression are most likely the causes for the changes in physiological functions. Specifically, we consider that an early regulation of transcription factors is indicative of regulation of gene expression. As we are working with glioblastoma cells (U118MG), one interest also includes how such changes in gene expression regulated by TSPO could affect tumorigenicity of these cells in this paradigm. For this purpose, we applied the TSPO ligand PK 11195 (25 µM) for several exposure times (15, 30, and 45 min, and 1, 3, and 24 h). Then, with the aid of microarray, we assayed changes in gene expression. The choice of 25 µM of PK 11195 is based on several previous studies that showed no adverse effects with this concentration, while it counteracts programmed cell death otherwise induced by various agents (e.g., [5,10,13,20,39]). Thus, in this paradigm PK 11195 at 25 µM only presents beneficial functional effects, including promotion of cell viability, and no disruptive lethal effects. In this study, apart from targeting the immediate question of regulation of nuclear gene expression by TSPO ligands, we also address the question whether modulation of gene expression may be associated with well-known TSPO functions. This was another reason to focus on PK 11195 at 25 µM, as by application of this concentration we expected to be better able to connect the present gene expression study with previous functional studies and their results that applied the same PK 11195 concentration. For these previous studies, dose response assays for application to U118MG cells were applied, which showed that 25 µM had optimal effects, without confounding side effects apparent with high doses, while lower doses in the paradigm of programmed cell death showed no effects by themselves (e.g., [5,10,13,20,44]). We also assayed whether functions predicted by the changes in gene expression could indeed also be actually observed in the present study (in particular functions that previously have not been considered part of the TSPO repertoire). Apart from the classical TSPO ligand PK 11195, we also assayed the effects of the more advanced TSPO ligand, 2-Cl-MGV-1 [13,20,45], to enhance the generality of the results.

While TSPO location in mitochondria has received a lot of attention, it is also known that TSPO can be present in perinuclear locations [7,46,47,48]. To investigate this further, we applied confocal microscopy to triple labeling of nuclei, mitochondria, and TSPO. Location restricted to the mitochondria would be supportive of TSPO’s role in mitochondria-to-nucleus signaling for regulation of nuclear gene expression. Intranuclear location could suggest a nuclear TSPO function, different from a mitochondrial TSPO function. Other intracellular locations could suggest a more elaborate involvement of TSPO in regulation of gene expression. As an extra, the study would show whether TSPO function associated with modulation of nuclear gene expression could correlate with known TSPO functions, and might possibly also reveal hitherto unknown TSPO functions.

## 2. Results

The presentation of the Results is divided into 5 parts: (1) PK 11195 effects on gene expression in general; (2) Potential effects of such gene expression changes on function, uncovered by pathway analysis; (3) Microscopic correlates at cellular and intracellular levels in association with changes in gene expression due to PK 11195 exposure; (4) Actual observations of phenotypic effects of PK 11195 exposure that were predicted by pathway analysis; (5) Comparison with effects of a more advanced TSPO ligand (2-Cl-MGV-1) on gene expression. For pathway analysis, we used the “Regulator Effects” analytic (IPA^®^) from Qiagen to gain more insights into the affected functions associated with the changes in gene expression, as outlined in the Methods.

### 2.1. PK 11195 Effects on Gene Expression in General

Exposure of U118MG cells to PK 11195 (25 µM) in our paradigm induced changes in gene expression of 1.5-fold or more of various genes in a time dependent fashion. We took a 1.5-fold change as a cut off to present changes in gene expression due to application of 25 µM of PK 11195 (Table 1). It was also taken into consideration whether groups of genes contributed to changes in functional pathways as detected by “Regulator Effects” analytic (IPA^®^). The complete raw data sets are filed with NCBI, names GSE77998 and “GSE85697” referenced in the section “Referenced Data Sets”. “GSE77998” indicates gene expression changes after 1, 3, and 24 h of PK 11195 (25 µM) exposure in our paradigm. “GSE85697” indicates gene expression changes after 15, 30, and 45 min of PK 11195 (25 µM) exposure in our paradigm.

As mentioned in the Introduction, we applied 25 µM of PK 11195 as this was an effective concentration determined in previous studies, in particular regarding regulation of programmed cell death, including modulation of mitochondrial membrane potential (ΔΨm), reactive oxygen species (ROS) generation, and cytochrome c release from mitochondria of U118MG cells. Importantly, this concentration also precludes confounding side effects [5]. Thereby, we assumed we would be able to associate our results with a previously established context [2,13]. First of all, we attained a general overview of gene expression changes following application of 25 µM of PK 11195, including magnitude and direction of changes in gene expression (Table 1). This provided a detailed time-course of changes in gene expression (Table 1).

After having determined changes in gene expression due to PK 11195 exposure in a time dependent fashion, we moved on to assay effects on function in general, in particular regarding induction of changes in gene expression and potentially subsequent functional effects (Table 2). Table 2 presents the gene symbols of the genes with significantly changed expression due to PK 11195 treatment, together with the general nature of the gene products (transcription factors, proteins, enzymes, and other products). Thus, as Table 1 provides a detailed time-course of changes in gene expression, Table 2 provides a detailed time course of related functional effects typically associated with the genes in question.

In short, starting at the left hand column of Table 2:

- Within 15 min, 20 genes significantly changed their expression rate in comparison to vehicle control, and 11 of them code for transcription factors, the other 9 code for proteins, enzymes, and other products.
- After 30 min (indicated in the second column) 14 genes changed significantly their expression rate from vehicle control. At this time point the number of genes coding for transcription factors is 6, the other 8 genes code for proteins, enzymes, and other products.
- After 45 min of PK 11195 exposure (indicated in the third column), 12 genes have their expression changed significantly from vehicle control, 5 of them coding for transcription factors, the other 7 genes code for proteins, enzymes, and other products.
- After 1 h (indicated in the fourth column), 25 genes significantly changed their expression rate, and 14 of them code for transcription factors, the other 11 code for proteins, enzymes, and other products.
- After 3 h (indicated in the fifth column), 14 genes changed significantly their expression rate from vehicle control. The number of genes coding for transcription factors at this time point is 6, the other 8 genes code for proteins, enzymes, and other products.
- After 24 h of PK 11195 exposure (indicated in the sixth column), 29 genes have their expression changed significantly from vehicle control. The number of genes coding for transcription factors is 6 after 24 h of PK 11195 exposure, the remaining majority of the genes (23 genes) codes for proteins, enzymes, and other products at this time point. Indeed, the biggest numbers of gene expression changes for proteins, enzymes, and other products is after 24 h.

As TSPO is well known to regulate programmed cell death, in Table 2 is also indicated with asterisks (*) which genes can be associated with programmed cell death. At every time point within the first hour of PK 11195 exposure, more than half of the genes with significantly changed gene expression are associated with programmed cell death. This number is smaller at 3 h and 24 h of PK 11195 exposure.

### 2.2. Implied Specific Functional Effects due to Gene Expression Changes Induced by Various PK 11195 Exposure Times

**Figure 1:**
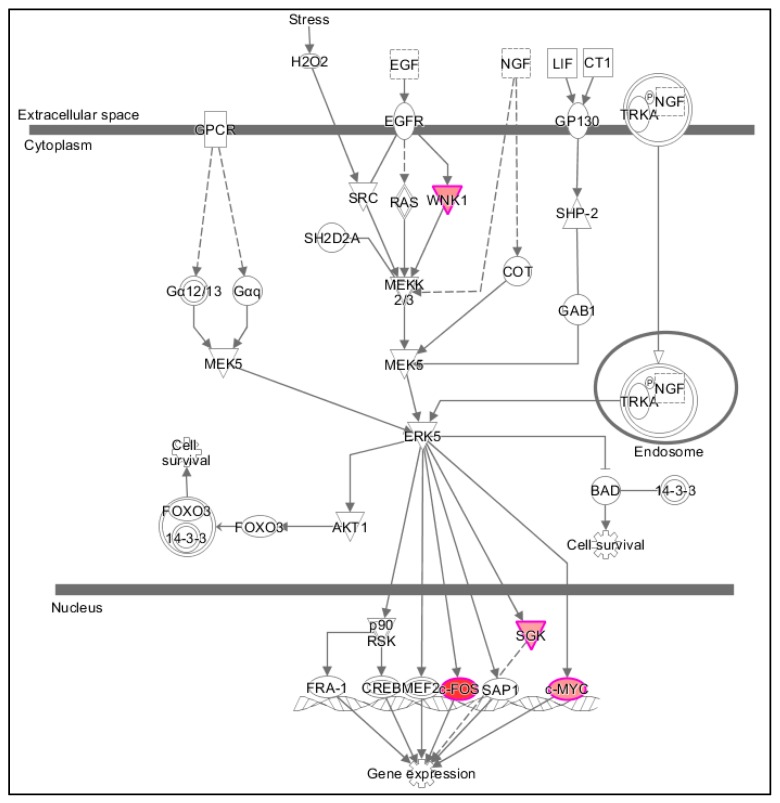
Specific elements of the canonical pathway for modulation of gene expression that are activated after 15 min of exposure of U118MG cells to 25 µM of PK 11195, as uncovered by “Regulator Effects” analytic (IPA^®^). The gene products of the genes *WNK1*, *FOS*, *SGK*, and *MYC* that are activated by the translocator protein (TSPO) ligand PK 11195 all are part of canonical pathways that converge on the final function of gene expression regulation. Furthermore, the statistically significant enhancements of expressions of the genes *WNK1*, *FOS*, *SGK*, and *MYC* all peak within one hour of exposure to PK 11195 (see Figure 2).

We used the “Regulator Effects” analytic (IPA^®^) to gain more insights into the potentially affected functions associated with the changes in gene expression. This showed that the products of at least some of the genes having their expression modulated after 15 min of exposure to PK 11195 are part of the canonical pathway for regulation of gene expression (Figure 1). These genes include *WNK1*, *FOS*, *SGK*, and *MYC.* Thus it appears that regulation or enabling of changes in nuclear gene expression in general indeed is one of TSPO’s functions.

**Figure 2:**
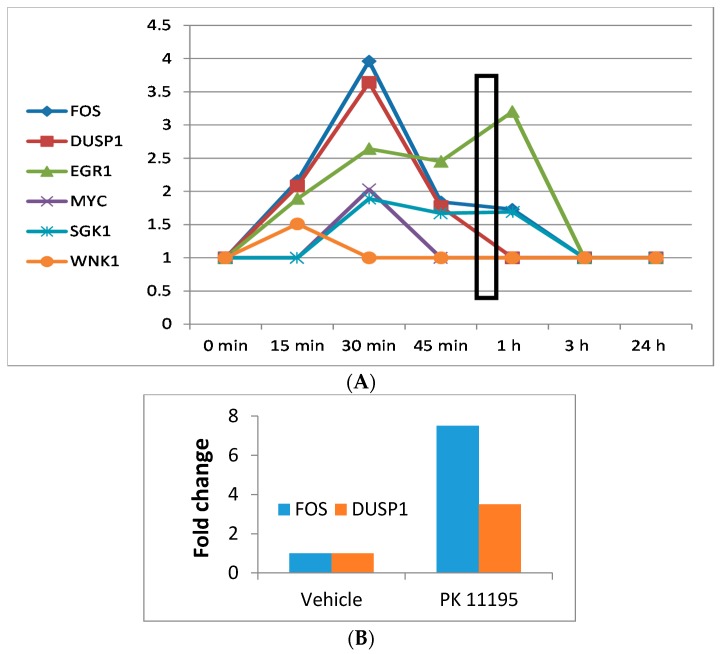
Effects of PK 11195 exposure on several immediate early genes of U118MG cells. (**A**) Time course of gene expression for gene products well known to take part in the initiation of modulation of gene expression assayed with microarray. These genes (*WNK1*, *FOS*, *DUSP1*, *EGR1*, *MYC*, *SGK1*) all present a peak of increased expression within half an hour of exposure of U118MG cells to 25 µM of PK 11195. As the data for 15, 30, and 45 min are obtained from one microarray and for 1, 3, and 24 h obtained from another microarray, a bar is placed between 45 and 60 min as a separation between the two. (Each micro array had its own untreated control as detailed in the Methods’ section); (**B)** Fold change (2^−ΔΔ*C*t^) of *FOS* and *DUSP1* expression after exposure to PK 11195 is 7.5 and 3.5, respectively, compared to untreated control (vehicle).

In Figure 2A, we established the time response curves of these 4 genes (*WNK1*, *FOS*, *SGK*, and *MYC*) that are highlighted in the canonical pathway for induction of gene expression changes presented in Figure 1, together with the 3 genes in Table 1 that showed most pronounced changes in gene expression (*FOS*, *DUSP1*, *EGR1*). *FOS*, *DUSP1*, and *EGR1* also are among the immediate early genes and transcription factors presented in Table 2. Note: the immediate early gene *FOS* is also part of the canonical pathway for induction of gene expression changes. Interestingly, the immediate early genes *FOS*, *DUSP1*, and *EGR1* consistently showed enhanced gene expression after 15, 30, and 45 min of exposure to PK 11195 (Figure 2A). Enhanced expression of *WNK1*, one of the initiators of the canonical pathway for gene expression, already peaks at 15 min. *FOS*, *MYC*, and *SGK* that are part of the intranuclear end of the canonical pathway for gene expression peak their expression at 30 min. *DUSP1* also peaks at 30 min. *EGR1* has a “small” peak at 30 min and a “high” peak at 60 min. Real time, reverse transciptase (RT)-PCR data showed increases in expression of *FOS* and *DUSP1* compared to control by 7.5 and 3.5-fold, respectively, corroborating the microarray data. In some detail, Table 3 gives the numbers of the C_t_’s of *FOS* and *DUSP1*, showing that RT-PCR also gives significant differences for the expression of these genes at 30 min of exposure as microarray also showed. Figure 2B gives the fold changes provided by the 2^−ΔΔ*C*t^ analysis of the *FOS* and *DUSP1* expression at 30 min measured with RT-PCR. Note regarding Figure 2B, since the samples vehicle and treated groups are not paired we are under obligation to use their averages for calculation of 2^−ΔΔ*C*t^ which thus gives only one data point for each gene presented by the columns in Figure 2C, and consequently no error bars. It is for this reason that we also provide Table 3, which does give the SD’s of the *C*_t_’s of *FOS* and *DUSP1* expression.

**Figure 3:**
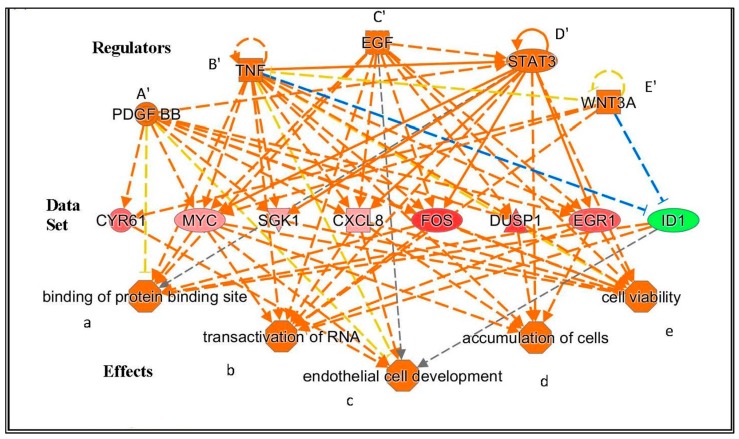
It presents the effects associated with changes in gene expression of 15 min of PK 11195 exposure, as determined with “Regulator Effects” analytic (in IPA^®^) from Qiagen as indicated in the figure (see also the Methods). The middle tier presents the genes showing significantly changed expression (“Data Set” in the middle tier). The “Effects” presented in the bottom tier indicate that, due to the significant changes in gene expression presented in the “Data Set”, the following general functions can be upregulated: (1) Binding of protein binding site; (2) Transactivation of RNA; (3) Endothelial cell development; (4) Accumulation of cells; and (5) Cell viability. The top tier presents the ‘Regulators’ known to be associated with these “Effects” and the genes of the “Data Set”. The arrows indicate the directions of the pathways, from Regulators, to Data Set, to Effect.

**Figure 4:**
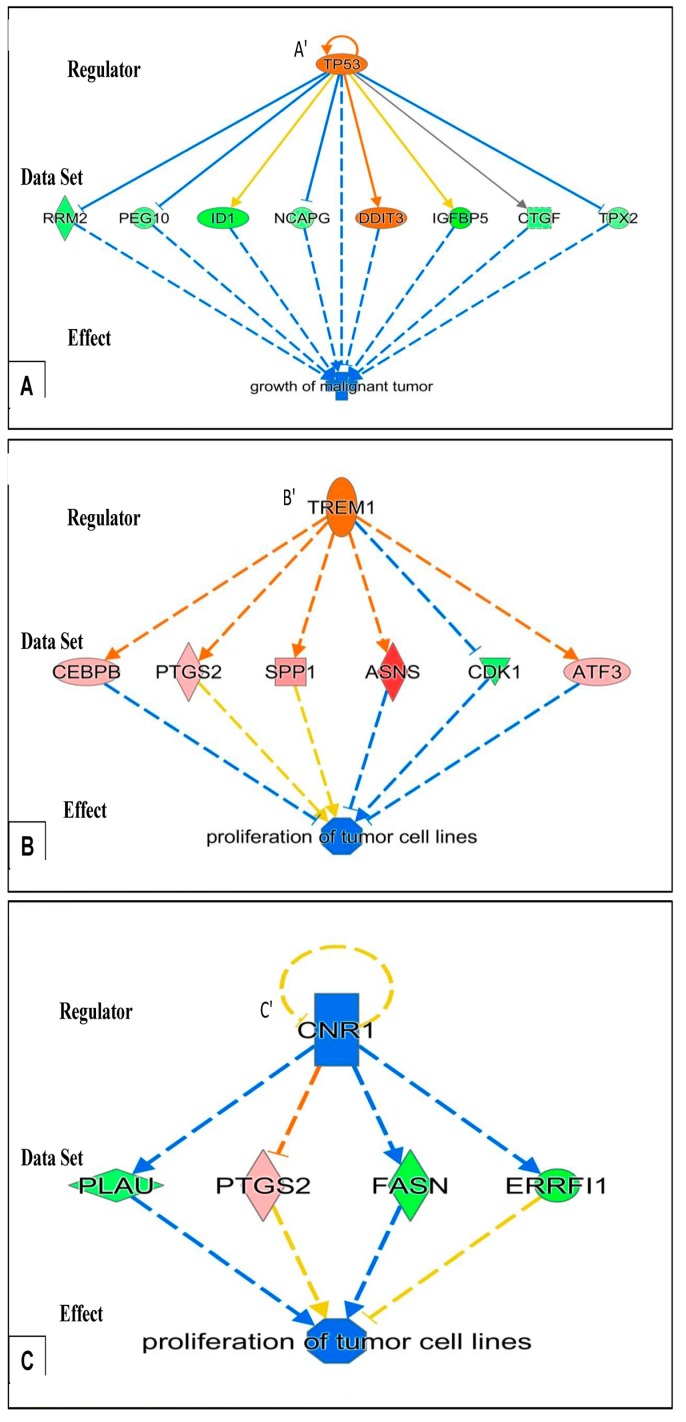
Potential effects on tumorigenicity due to gene expression following 24 h of exposure of U118MG cells to PK 11195 (25 µM). As determined with “Regulator Effects” analytic (IPA^®^) from Qiagen as indicated in the figure (see also the Methods)., in (**A**–**C**), individual “Regulators” (given in the upper tiers) are related to specific groups of genes with significantly changed expression (“Data Sets” given in the middle tiers), together with their particular downstream functions (“Effects” in the bottom tiers), namely, suppression of growth of malignant tumor (in (**A**) and suppression of proliferation of tumor cell lines (in (**B**,**C**). Color coding: pink/orange = upregulated, blue/green = down regulated. The configurations in seen in (**A**–**C**) can be considered assemblies. The arrows indicate the directions of the pathways, from Regulators, to Data Set, to Effect.

**Figure 5:**
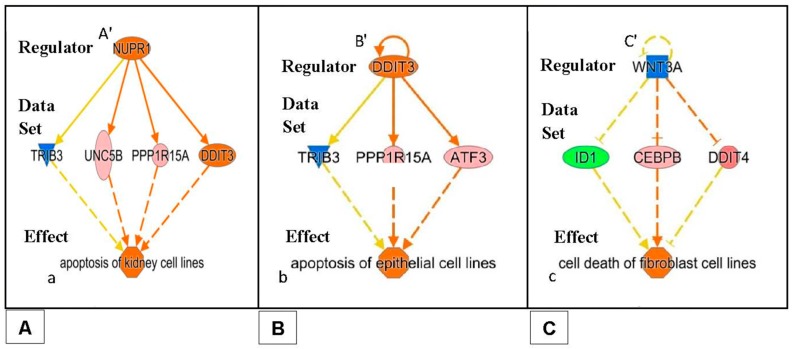
Potential effects on programmed cell death due to gene expression following 24 h of exposure of U118MG cells to PK 11195 (25 µM). As analyzed with Regulator Effects analytic (IPA^®^) from Qiagen as indicated in the figure (see also the Methods), in A,B,C, individual “Regulators” (given in the upper tiers) are related to specific groups of genes with significantly changed expression (“Data Sets” given in the middle tiers), together with their particular downstream functions (“Effects” in the bottom tiers), namely, stimulation of apoptosis of kidney cell lines (in (**A**), stimulation of apoptosis of epithelial cell lines (in (**B**)), stimulation of cell death of fibroblast cell lines (in (**C**). Each mentioned separate set can be considered an assembly of pathways running from 1 or few Regulators via a number of genes to affect not more than 1 or 2 specific functions. Color coding: pink/orange = upregulated, blue/green = down regulated. The configurations in seen in (**A**–**C**) can be considered assemblies. The arrows indicate the directions of the pathways, from Regulators, to Data Set, to Effect.

The postulated functional effects due to the gene expression changes seen at the different time points as provided by “Regulator Effects” analytic (IPA^®^) are given in Table 4. As the detailed presentations of the outcomes of these analyses are very elaborate for each of the time points, they are provided in the supplementary materials. In the body of the results, we have given examples of the results of this analysis for 15 min in Figure 1, Figure 2 and Figure 3, and for 24 h in Figure 4 and Figure 5, as at these time points the outcome of the analysis is relatively straightforward, and insightful. For completeness, the results of the analysis for all the time points 15, 30, 45, and 60 min, and of 3 and 24 h are provided in the supplementary files, and presented summarily here in the Results section, e.g., in Table 4.

The functional effects displayed in Table 4 are provided by the downstream components (“Effects”), given by the application of “Regulator Effects” analytic (in IPA^®^) to our data. This gives insights into potential phenotypic and physiological effects that may result from the changes in gene expression due to exposure of U118MG cells to 25 µM of PK 11195, including the time-course of the phenotypic and physiological changes. The gene expression data for each time point, provided by the microarray assays, is given in the “Data Sets” of the figures that present the “Regulator Effect”. In the body of the text, these figures are given only for 15 min and 24 h of PK 11195 exposure (Figure 3, Figure 4 and Figure 5). The “Regulators” and “Effects” present what is known regarding upstream regulation and downstream effects of these genes. For the complete overview of the time points of 15, 30, 45 min, 1, 3, and 24 h, providing for the time course of these events, see the appendices. These appendices present “Data Sets”, “Regulators”, and “Effects” for each time point.

As examples in the body of the Results, the “Effects” are presented in the bottom tiers of Figure 3, Figure 4 and Figure 5 for PK 11195 exposures of 15 min and 24 h. The “Effects” present the functional, phenotypic, and disease related effects that are known to be under control of the genes with changed expression in our study. The middle tiers of these Figure 3, Figure 4 and Figure 5 present the genes with changed expression determined in our study (the “Data Sets”) as exemplified by “Regulator Effects” analytic (IPA^®^). The “Regulators” presented in the top tiers of these diagrams of Figure 3, Figure 4 and Figure 5 are factors (genes, RNAs, proteins) that reportedly can modulate expression of the genes of our “Data Sets”. As mentioned, the appendices also show the assayed time points between 15 min and 24 h.

Figure 3, Figure 4 and Figure 5 show that in these paradigms individual “Regulators” can modulate genes’ expression from clusters of various sets of genes. A set of genes that can be modulated by one “Regulator” that affects one particular “Effect” can be considered an assembly. Such assemblies can be seen in Figure 4 and Figure 5 (describing gene expression effects for 24 h of PK 11195 application). Interacting and overlapping assemblies can be considered super-assemblies. One such super-assembly is presented in Figure 3 (describing gene expression effects for 15 min of PK 11195 application, which actually is a relatively simple super-assembly). In the appendices, the super-assemblies induced by 15, 30, 45, and 60 min, and 3 and 24 h of PK 11195 exposures are given with more additional details. The assemblies and super-assemblies indicate to us that the functions affected are not based on the changes in expression of just one single gene, but based on several genes. This indicates that the postulations of changes in functions are robust. General terms used for these phenomena providing robustness regarding regulation are “redundancy” and “degeneracy” as exemplified in the Discussion [49,50,51]. We favored the application of ‘Regulator Effects’ analytic (IPA^®^), and the unadulterated presentation of its outcomes, to avoid selective bias potentially introduced by preconceived interpretations of what the effects should be. This way of gene expression analysis is particularly useful for TSPO research as it is well known that TSPO affects a broad variety of functions [1,2,13]. The functional effects are presented below, in a temporal fashion:

- Regarding functional effects at 15 min, in addition to upregulation of gene expression, in general, several functions appeared to be affected. In particular, binding of protein binding site, transactivation of RNA, cell development, cell viability, and accumulation of cells (Table 4, Figure 3). As seen in Figure 3, 15 min of PK 11195 activates a super-assembly including 5 Regulators, 8 genes, and 5 Effects. In Figure 1 and Figure 2 is shown that PK 11195 application for 15 min affects the canonical pathway for regulation of gene expression.
- Functional effects at 30 min appeared to be more varied than at 15 min of PK 11195 exposure. For simplification, these effects can be classified as: binding of protein binding site, cell division and proliferation, cell viability, metabolism, cell differentiation, cell motility, tumorigenicity, and tissue inflammation. These functions are listed in more detail in Table 4. The super-assembly that can be distinguished at 30 min is elaborate and includes 26 Regulators, 19 genes, and 15 Effects. This super-assembly is provided in the supplementary files.
- After 45 min, cell differentiation effects appeared to be the core functional aspect of the gene expression changes, as well as angiogenesis, proliferation, migration, and cell growth (Table 4). The super-assembly seen at 45 min is relatively small, 9 Regulators, 9 genes, and 3 Effects (provided in the supplementary files). Both the 30 and 45 min of PK 11195 exposures caused gene expression changes associated with the canonical pathway for angiogenesis, also provided in the supplementary files.
- After 1 h, the functional effects in general appeared to include: upregulation of cell cycle, proliferation, cell differentiation, cell viability, and tumorigenesis, but also programmed cell death. These functions are listed in more detail in Table 4. The super-assembly seen at 1 h includes 19 Regulators, 29 genes, and 12 Effects (provided in the supplementary files).
- After 3 h, the general effect due to changes in expression of the various genes after exposure of U118MG cells to 25 µM of PK 11195 appears to imply a less tumorigenic phenotype. The majority of the ‘Effects’ of 3 h of PK 11195 exposure can be classified as down regulation. This down regulation relates to (1) Migration; (2) Inflammatory response; (3) Proliferation, (4) Development, including cell differentiation; (5) Cell viability; and (6) Tumorigenesis. These ‘Effects’ after 3 h are virtually the opposite from those seen after the shorter PK 11195 exposures. These functions are listed in more detail in Table 3. In contrast, programmed cell death is still upregulated, as was also seen after 1 h of PK 11195 exposure. These functions are listed in more detail in Table 4. The super-assembly activated by 3 h of PK 11195 exposure includes 23 Regulators, 30 genes, and 18 Effects, presented in the supplementary files.
- After 24 h of PK 11195 exposure, pathway analysis with the “Regulator Effects” analytic (IPA^®^) indicated that due to the significant changes in gene expression only the following general function is down-regulated: tumorigenicity (Figure 4). Several separate pathways were revealed regarding tumorigenicity, each one including just one “Regulator” and a small set of genes forming the “Data Set” (Figure 4A–C). Additional figures in the Supplementary Materials give additional, somewhat more complicated information, i.e., 2 or 3 “Regulators” together modulating ‘Data Sets’ of a dozen to several dozen genes (Supplementary Materials). These figures also impinge on the general theme of reduced tumorigenicity. Thus, after 24 h of PK 11195 exposure no extensive super-assembly was recognized, but several independent assemblies downregulating several aspects of tumorigenicity and upregulation of programmed cell death (Figure 4 and Figure 5).

Regarding programmed cell death , with all PK 11195 exposure times, “Regulator Effects” analytic (IPA^®^) of Qiagen indicated significant changes in gene expression associated with programmed cell death (Table 2).

Taking the whole time sequence of responses to PK 11195 into account, it appears that during the first hour upregulation of tumorigenic responses prevails. At 60 min pro-aptotic gene products become relevant and remain so till at least 24 h after PK 11195 exposure. Furthermore, at 3 and 24 h of PK 11195 exposure, down regulation of tumorigenic responses becomes predominant. This response sequence may be interpreted as homeostatic.

### 2.3. Microscopic Correlates at Cellular and Intracellular Levels in Association with Changes in Gene Expression due to PK 11195 Exposure

**Figure 6:**
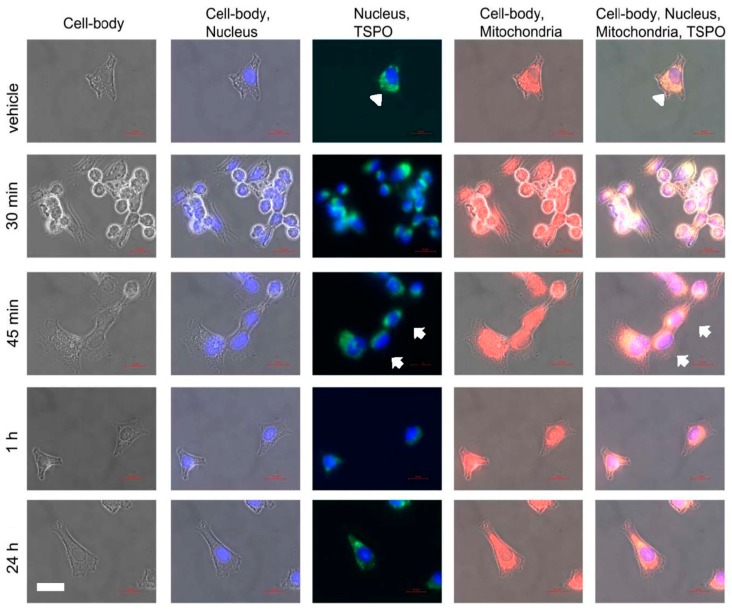
Phase contrast microscopic images of U118MG cells labeled for the cell nucleus (DAPI), the mitochondria (Mitotracker Red), and TSPO (immunocytological labeling). The images are indicative of morphological changes due to exposure to the TSPO ligand PK 11195, in association with intracellular TSPO location. In general, as described in the text, the main points are that TSPO does not appear in the cell nuclei, but are always co-localized with mitochondria. The arrow-heads indicate labeling of mitochondria and TSPO evenly distributed throughout the cytoplasm in the vehicle row. In the 45 min row, the arrows point at mitochondria with co-localized TSPO that are present relatively close to the cell nuclei. A more detailed description of the figure follows here: The rows present exposure times to PK 11195 (25 µM), from top to bottom: vehicle (i.e., no exposure), 30, 45 min, 1, and 24 h. In each row from column to column the same cells are shown. Phase contrast micrographs are presented in the first column. The second column shows cell nuclei (stained blue with the aid of DAPI) within the phase contrast images. The third column shows TSPO immune labeling (Alexa fluor^R^ 488, i.e., green) together with the DAPI stained nuclei. Most importantly, the images in the third column show that TSPO labeling is not within the cell nuclei, but in other intracellular areas and cell organelles. In particular, there is no double-labeling of TSPO and DAPI signal. The fourth column shows mitochondrial labeling in the cells outlined by phase contrast. Here it becomes clear that the TSPO labeling in the third column covers intracellular areas occupied by mitochondria. The fifth column shows the results of all signals for the cells in question combined. This presents merged double labeling for mitochondria and TSPO (white to yellow), while phase contrast and nuclear stain is applied for orientation. The DAPI stained nuclei appear purple here due to interference. As a control for this, omission of TSPO labeling i.e., by omitting the primary anti-TSPO anti-body, results in the same nuclear stain (purple) when applying the same microscopic conditions (not shown). As a general remark, omission of TSPO antibody completely prevented the immunocytochemical labeling for TSPO (not shown). The scale bar of 20 µm in the lower left corner of the figure is for all the micrographs in this plate.

**Figure 7:**
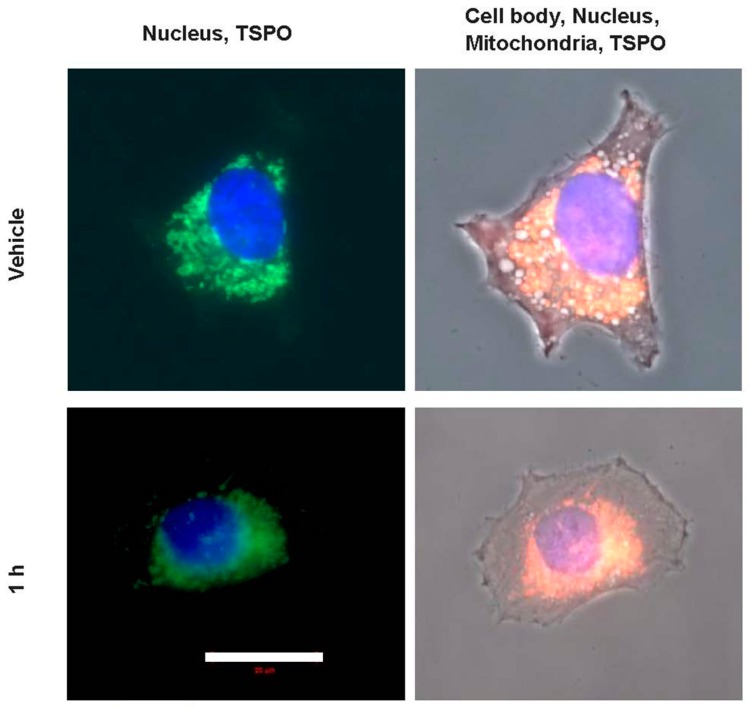
Phase contrast microscopic images of U118MG cells labeled for the intracellular location of TSPO. In the left hand images, cells are only viewed for labeling of the cell nucleus (DAPI) and of TSPO (immunocytological labeling). In the right hand images, cells are viewed for labeling of the cell nucleus (DAPI), TSPO (immunocytological labeling), and the mitochondria (Mitotracker Red), within the cells outlined by phase contrast. While TSPO labeling can appear in the same area of view as the cell nucleus, double labeling of TSPO and nucleus does not occur (left hand images). In the right hand images it can be seen that the TSPO labeling overlaying the nucleus double labels with mitochondria, resulting in a yellow-whitish stain. The main points are that TSPO does not appear in the cell nuclei, but are always co-localized with mitochondria. In the vehicle exposed cells (top two images), labeling of mitochondria and TSPO is evenly distributed throughout the cytoplasm, almost completely filling out the cell body. In the cells exposed for 1 h to PK 11195, labeling of mitochondria and TSPO is condensed toward the cell nucleus, with TSPO—mitochondria double labeling appearing relatively close to the nucleus. Toward the periphery of the cell body a relative broad area is devoid of mitochondrial as well as TSPO labeling. The scale bar in the lower left micrograph of the figure is of 20 µm for all the micrographs in the plate.

To see whether the changes in gene expression due to PK 11195 exposures, seen at the different time points in the present study, may be associated with changes in TSPO location, confocal microscopy was applied (Figure 6). A priori, we had expected to see TSPO expression appearing in the cell nucleus after PK 11195 exposure, in association with the apparent modulation of gene expression. Actually, we did not expect to see changes in gene expression before 24 h. Thus, we expected changes in gene expression to come only after 24 h of PK 11195 exposure, potentially even associated with a shift in TSPO location. However, as mentioned above, changes in gene expression determined with microarray were already apparent within 15 min of PK 11195 application. As the gene expression assay, also our microscopic study started with the time point of 15 min, and included 30, and 45 min, 1, 3, and 24 h. A general overview of the results is given in Figure 6. For simplification, Figure 6 is restricted to the time points of 0 min exposure (vehicle control), 30, 45 min, 1, and 24 h. We found that in all instances, i.e., in vehicle control as well as at all of the time points for PK 11195 exposed cells, immunocytological TSPO labeling appears to be restricted to the cytosolic compartment of the cell, including the mitochondria (labeled with Mitotracker red). To emphasize, TSPO labeling does not double-label with DAPI labeling of the cell nuclei, as can be seen clearly in the columns named “Nucleus, TSPO” of Figure 6 and Figure 7.

We think is noteworthy to mention that we encountered a potentially interesting additional observation. At every time point of PK 11195 exposure, see for example the column of Figure 6 named “Nucleus, TSPO”, dense labeling of TSPO in relative proximity to the nucleus can be recognized in at least several of the PK 11195 treated cells (where this is most clear it is marked with white arrows). Such dense TSPO labeling can even become visible as “caps” adjacent to the cell nuclei. This is not apparent at all in cells not treated with PK 11195. The even spread of mitochondria labeled with TSPO throughout the cell body of vehicle control cells is indicated with white arrow heads in the top row of Figure 6. Examples of these phenomena are given as expanded images in Figure 7. For Figure 7 we chose the time points of 0 min and 1 h (i.e., with and without PK 11195 exposure), with the representations of: (1) labeling for TSPO, mitochondria, cell nuclei, and phase contrast for the cell outline to achieve a relatively complete overview of TSPO distribution inside the cell body; (2) labeling for TSPO and cell nuclei indicating the lack of TSPO labeling inside the cell nuclei, in unexposed as well as PK 11195 exposed cells. Thus, after PK 11195 exposure, TSPO labeling, as well as mitochondrial labeling, appears relatively condensed toward the nucleus, leaving more peripheral cellular regions free from this labeling , while in unexposed cells such labeling appears evenly spread over the cytosol. Note, TSPO labeling only double-labels with mitotracker red (labeling for mitochondria), never with DAPI (labeling for cell nuclei), even when TSPO labeling is in the same area of view as the cell nucleus. Figure 6 provides a more differentiated overview, including changes in the morphology of cells during the exposures to PK 11195. See a more detailed description of Figure 6 further below.

**Figure 8:**
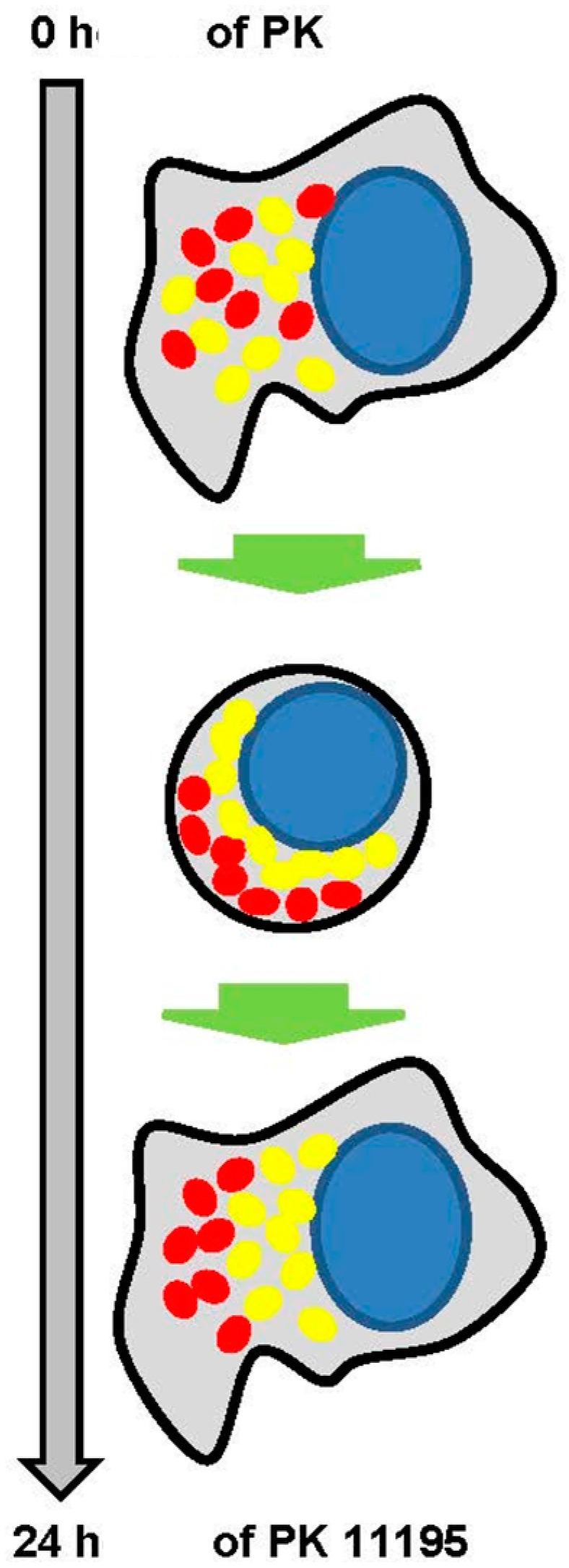
Scheme of observations presented in Figure 6 and Figure 7. In vehicle control cells (i.e., 0 h of PK 11195 exposure) mitochondria with TSPO (yellow) are spread throughout the cytosol (grey). Shortly after PK 11195 exposure the cell bodies contract, become roundish, and mitochondria with TSPO appear to become more condensed toward the cell nuclei (blue). After 24 h of PK 11195 exposure the cell bodies regain their original polygonal shapes. Nonetheless, after these 24 h, mitochondria with TSPO still remain congregated in areas relatively close to the cell nucleus. Mitochondria not displaying TSPO signal are indicated with (red).

The scheme of Figure 8 presents some core observations of cell morphology presented in Figure 6 and Figure 7. In particular, in vehicle control (0 h) TSPO labeling appears to be evenly spread throughout the cytoplasm together with the mitochondria (top of Figure 8). As illustrated in the diagram of Figure 8, commonly, in cells exposed to PK 11195, mitochondria with dense TSPO labeling can be seen relatively close to the cell nuclei (from halfway down Figure 8 to the bottom of Figure 8). Also the basic time dependent cell body shapes (round and polygonal) are diagrammatized in Figure 8. At the top of the diagram of Figure 8, the cells (not exposed to PK 11195) are polygonal. One step lower in the diagram of Figure 8, rounded cells are presented (i.e., cells exposed to PK 11195 for 15 and 30 min). From the time point of 45 min of exposure to PK 11195, the U118MG cells start to disperse from the clusters and revert to their typical polygonal and elongated shapes (bottom of the diagram of Figure 8).

These observations are given here in more detail as a guide through the presentation of Figure 6. Going down row by row with the sequential time responses to PK 11195 exposure in Figure 6, it can be seen in the top row, or row 1 (named vehicle i.e., zero time exposure to PK 11195), that TSPO labeling is relatively evenly spread over the intracellular areas covered by mitochondria, without distinct areas of enhanced signal. This, for example, is indicated by arrow heads at the intersections of row 1 with columns 3 and 5. The cells themselves are polygonal in shape and evenly distributed over the coverslip, i.e., without clustering. In row 2 (30 min exposure to PK 11195) the cells have congregated to form clusters and frequently present roundish shapes, while the mitochondria with their TSPO appear to be condensed toward the cell nucleus (presented at the intersections with columns 3 and 5). The same is true for 15 min of PK 11195 exposure (not shown). In row 3 (45 min exposure to TSPO), the cells appear to revert to their original morphology again (as in row 1). Nonetheless, mitochondria double labeled with TSPO remain visible relatively close to the cell nuclei. This phenomenon is indicated by arrows in the images of columns 3 and 5 intersecting with row 3. Note well that this phenomenon of distinct TSPO signal present in mitochondria relatively close to the nuclei is observed at all time points of PK 11195 exposure, i.e., in Figure 6 in all rows from row 2 to row 5, but not in row 1 (which is vehicle control). In rows 4 and 5 (respectively 1 and 24 h of PK 11195 exposure), the cells continue to revert to their original morphology and at 24 h have become indistinguishable from vehicle exposed U118MG cells (row 1). The same is true for 3 h of PK 11195 exposure (not shown). The basic observations are schematized in a relatively simple manner in Figure 8, presenting that TSPO only occurs in the mitochondria in this paradigm, never in the cell nucleus.

The phenomena presented in Figure 6, Figure 7 and Figure 8, were observed in two separate experiments, where each condition was provided in 3 wells, and where at least 10 cells, or 10 clusters of cells from each well were photographed. At present we cannot say what would be the cause, mechanism, or functional significance for the observed enhanced TSPO signal in the relative proximity of the cell nucleus in PK 11195 exposed cells.

### 2.4. Actual Phenotypic Effects of PK 11195 Exposure That Were Predicted by Pathway Analysis

Several functional effects implicated by the changes in gene expression described above could indeed be discerned by simple microscopic observations in our present study (Figure 6, Figure 7 and Figure 8). For example, functional effects predicted by the changes in gene expression at 15 min included accumulation of cells, cell viability, and cell development (Table 3). Microscopically, accumulation of cells after PK 11195 exposures was very conspicuous (Figure 6). This was particularly true at 15 min and 30 min, and could still be discerned at 45 min (Figure 6). Note: accumulation of cells due to PK 11195 exposure has not been described before. Thus, we could microscopically observe the cell accumulation that was predicted by our gene expression assays. At 30 and 45 min, functional effects of gene expression changes also implicated cell migration (Table 3). The congregation and segregation of the cells under microscopic observations matched the cell migration effects implicated by the gene expression analysis (Figure 6). Possibly the microtubule dynamics presented as an ‘Effect’ at 30 min can be associated with changes in cell body shape observed microscopically (Figure 6 and Figure 7). These cell soma changes may be related to other functions, such as migration, adhesion, cell division, etc., which at present cannot be decided. After 1 h, cell migration no longer did appear to be a function implicated by gene expression changes, and after 3 h such effect appeared to be reversed. Also under the microscope, signs of cell migration could no longer be discerned after one hour of PK 11195 exposure. Regulator Effects analytic (IPA^®^) of Qiagen also suggested cell viability effects, as uncovered at all time points. Indeed, previous experimental studies have presented PK 11195 at 25 µM to be a cell protective agent (e.g., [5]), although at that time gene expression effects were not considered.

**Figure 9:**
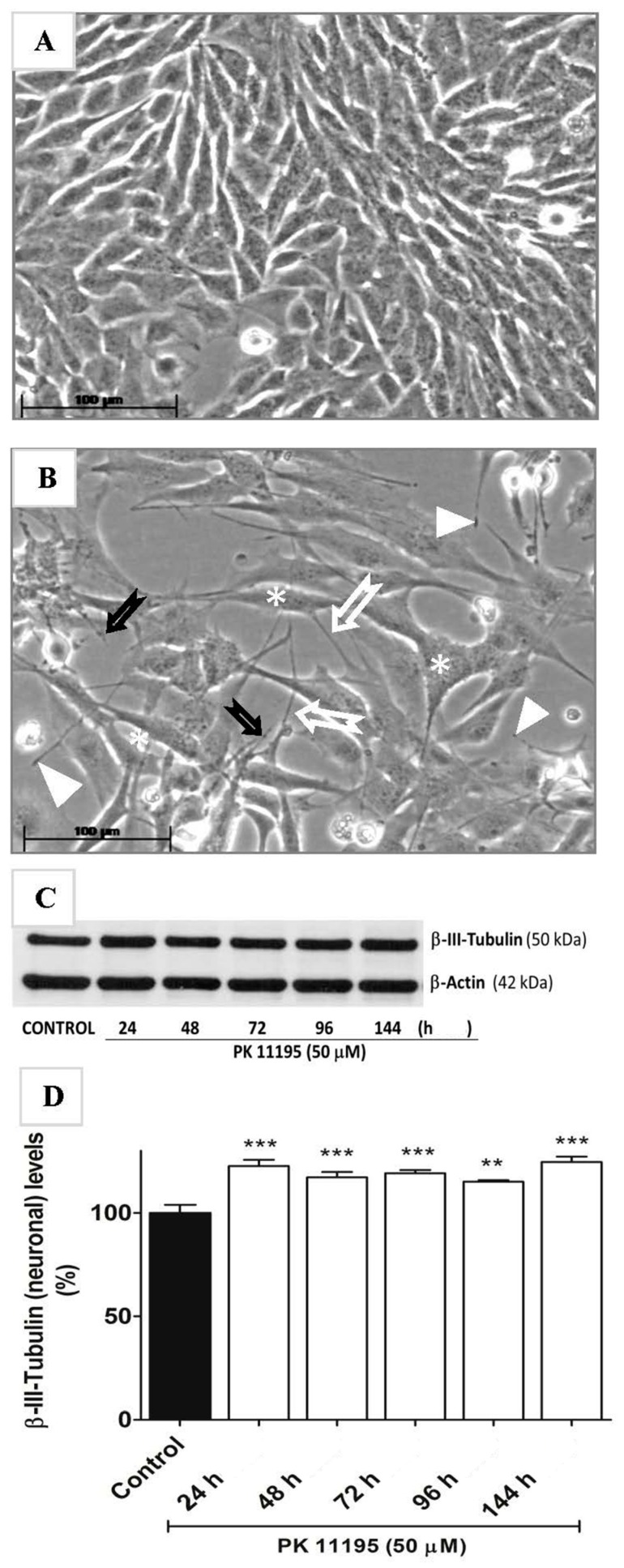
PK 11195 induced neuronal differentiation of rat PC12 cells. (**A**) Undifferentiated vehicle control cells; (**B**) Neuronal differentiation due to PK 11195 (50 µM), including differentiating cells (white asterisks), neurite outgrowth (white arrows), growth cones (white arrowheads), and varicosities (black arrows); (**C**) Representative western blot showing elevated β-III-tubulin expression in rat PC12 cells after exposure times of 24, 48, 72, 96, and 144 h to PK 11195 (50 µM). β-actin is the loading control; (**D**) Bar chart of Means ± SEM (*n* = 3) of the relative densities of the blot bands of β-III-Tubulin labeling in C (arbitrary units as% of control. Control = vehicle treated cells. ** *p* < 0.01, *** *p* < 0.001. (In (**A**,**B**); bars: 100 µm).

Another major effect of gene expression changes implied at different time points was cell development and differentiation, even neuronal development and differentiation (from the time point of 15 min till the time point of 3 h) (Table 3). The suggestion that exposure to PK 11195 can lead to development and differentiation of neurons was a surprise, as U118MG cells are not considered neuronal. We proceeded to test this potentiality in PC12 cells, which can be considered neuronal progenitor cells. With previous studies we had shown that other, more advanced TSPO ligands can induce neuronal differentiation from these neural progenitor cells, including enhanced tubulin expression [46]. Indeed also in this study, PK 11195, without any additional treatment, just by itself, could very clearly induce neuronal differentiation, including development of neurites and increased expression of tubulin (Figure 9). The observed tubulin expression may be associated with microtubule dynamics also observed with our gene expression assays.

As TSPO is well known to affect programmed cell death, we paid special attention to this issue. PK 11195 exposure appeared to have a time dependent effect on gene expression related to programmed cell death, as indicated by asterisks (*) next to the genes in question in Table 2: After 15 min, 11 out of 20 genes; after 30 min, 9 out of 14 genes; after 45 min, 8 out of 12 genes; after 60 min, 17 out of 25 genes; after 3 h, 6 out of 14 genes; and after 24 h, 9 out of 29 genes. Thus, at all time points within 60 min more than half of the genes changing expression are related to programmed cell death. After 3 h it is less than half, and after 24 h less than one third (Table 2).

In general, apart from the functions discovered in the present study (e.g., accumulation of cells, neuron differentiation), the functions revealed by Regulator Effects analytic; IPA^®^ include numerous well established TSPO functions (see Discussion and Introduction).

### 2.5. Effects of TSPO Ligands Other Than PK 11195 on Gene Expression

We chose PK 11195 to test potential induction of gene expression via TSPO, as PK 11195 is a classical, well studied TSPO ligand, and the results can be in a relatively facile way incorporated in the accumulated body of knowledge. Moreover, effects of 25 µm of PK 11195 are comparable to TSPO knockdown (e.g., [32,39]). However, we also wanted to determine whether more advanced TSPO ligands also present modulation of gene expression. To investigate this we applied different concentrations of a recently developed TSPO ligand 2-Cl-MGV-1 [13,20,46]. For the study of potential 2-Cl-MGV-1 effects, we used the same microarray assay method as for PK 11195. We assumed that this would give an indication of the specificity of the effects of PK 11195 as a TSPO ligand. We applied 1 h of exposure as this was an effective time period as demonstrated for changes in gene expression following PK 11195 application. We applied concentrations at the same height or higher than we applied for PK 11195 (i.e., 25, 50, and 100 µM), because the affinity of 2-Cl-MGV-1 is much lower than of PK 11195 (~240-fold) [46]. Furthermore, at 50 and 100 µM 2-Cl-MGV-1 does not show the confounding side effects that PK 11195 shows at these concentrations [5,20]. In this paradigm 2-Cl-MGV-1 significantly affected a small number of genes primarily related to gene expression regulation. Table 5 shows the genes that are regulated by 2-Cl-MGV-1, namely: *FOS*, *ZFP36*, *DUSP1*, *TUFT1*, and *ID2*, and This Table 5 shows that the concentrations of 50 and 100 µM 2-Cl-MGV-1 have robust effects on the expression of these genes. A cut off of 1.5 as applied for PK 11195, showed changes due to application of 50 and 100 µM of 2-Cl-MGV-1. Importantly, all the genes as seen in Table 5 are among the genes of which their expression is most affected by PK 11195 (see Table 1). Thus, effects on these genes by the TSPO ligands we applied in this paradigm appear to be quite specifically related to control of overall gene expression.

### 2.6. General Observations

The TSPO ligand PK 11195, significantly and specifically, within 15 min induces changes in gene expression. These gene expression changes are in accord with actual phenotypic and functional changes. Microscopic observations imply mitochondrial TSPO in these phenomena.

## 3. Discussion

To answer the question whether nuclear gene expression can be modulated via the mitochondrial TSPO: (i) we first of all studied the effects of the classical TSPO ligand PK 11195 on gene expression in general, in a time dependent fashion. (ii) We then applied pathway analysis to predict potential functional implications. (iii) We also designed microscopic studies to study whether and how TSPO location could be associated with modulation of gene expression i.e., to determine whether (preferred) location of TSPO could change between mitochondria and nucleus. (iv) Microscopy was also applied to study whether functions were actually modulated as predicted by our pathway analysis. (v) Finally, comparisons with effects on gene expression by a more advanced TSPO ligand 2-Cl-MGV-1 were undertaken.

The early effect on gene expression (at 15 min of PK 11195 exposure) is an important indication provided by the present study that TSPO may be an integral part of a pathway for regulation of nuclear gene expression. PK 11195 is a specific TSPO ligand, known to affect numerous TSPO functions, similar to the effects of TSPO knockdown by genetic manipulation [14,35,37,44]. The effects seen on gene expression in the present study within this relatively short time of 15 min precede physiological changes (in particular phenotypic changes) typically caused by TSPO ligands. In general, various cellular functional changes in diverse cell cultures, including U118MG cells, typically are detected only after 24 h of TSPO ligand treatment [5,14,35,37,52]. A detailed time response study by Costa et al. [40], applying the covalent TSPO ligand irDE-MPIGA to GBM cells, showed that it takes more than 90 min for irDE-MPIGA (25 nM) to irreversibly saturate all TSPO binding sites; 3 h after ligand application ΔΨm collapse was observed; 6 h after ligand application externalization of phosphatidylserine was observed and cell viability was reduced. After 24 h, de-novo TSPO synthesis was observed [40]. The present study shows that modulation of gene expression in U118MG cells due to PK 11195 occurs hours before these previously reported physiological and phenotypic changes [5,14,35,37,40,52]. Thus, the changes in gene expression appear to be the cause rather than the effect of such changes. Moreover, Ingenuity pathway analysis (IPA^®^) also indicated that the first canonical pathway affected in our paradigm, i.e., at 15 min, was restricted to regulation of overall gene expression. Furthermore, our results showed that several key immediate early genes and transcription factor present a pronounced peak in increased expression around 30 min of PK 11195 exposure. These key immediate early genes and transcription factor are *WNK1*, *SGK*, *FOS*, *DUSP1*, and *EGR1*. As an additional test, also required for follow up studies, we also have applied real time RT-PCR according to standard methods to determine changes in expression of the immediate early genes *FOS* and *DUSP1*. With this RT-PCR assay, we found that relative expression of *FOS* and *DUSP1* in samples of PK 11195 treated cells was increased in comparison to vehicle treated cells. In numbers, relative concentration of *FOS* in cells treated for 30 min by PK 11195 was 7.5 compared to vehicle control, and for *DUSP1* this relative concentration was 3.5. This considerable increase in gene expression seen with real time RT-PCR basically corroborates the microarray results of 30 min exposure to PK 11195. We selected *FOS* and *DUSP1* for this purpose, as their peak expression at 30 min with microarray is one of the main indicators that the classical TSPO ligand PK 11195 can modulate nuclear gene expression, including the canonical pathway for regulation of overall gene expression, as exemplified in Figure 1 and Figure 2. Also application of the TSPO ligand 2-Cl-MGV-1 for one hour to U118MG cells was indicative of effects on gene expression, including immediate early genes and transcription factors: *FOS*, *ZFP36*, *DUSP1*, *TUFT1*, and *ID2*. Thus, our experiments indicate that regulation of gene expression by different TSPO ligands appears to be a reproducible phenomenon. Subsequently over the course of 24 h of PK 11195 exposure the effects on gene expression appeared to present a very dynamic process, i.e., apart from the genes directly involved in regulation of gene expression, various genes affecting various functions over this time period were affected. Finally, after 24 h, the gene expression in this glioblastoma cell line of U118MG was regulated such that an anti-tumorigenic effect became most evident, including promotion of programmed cell death. Furthermore, changes in gene expression observed after 3 h appeared to promote effects that counteract effects determined at shorter time periods, thus being suggestive of a homeostatic effect.

Regarding the choice of the concentration of PK 11195 applied, with several previous studies we found that PK 11195 at a concentration of 25 µM could optimally prevent programmed cell death otherwise induced by various agents (ErPC3, glutamate, NO, CoCl_2_). To emphasize, these effects 25 µM of PK 11195 are similar to the effects of TSPO knockdown. For a review of these studies see [10]. In the Kugler et al. study [5] it was determined that lower concentrations from 1 µM down to 1 nM had no effect at all on programmed cell death induced by ErPC3. Just by itself, PK 11195 concentrations of 25 µM and lower (as low as 1 nM) had no effect at all i.e., appeared neutral. Higher PK 11195 concentrations (in particular higher than 50 µM) presented increasing lethal effects on their own, an effect that is well known [5,8]. Thus, this indicated to us that 25 µM of PK 11195 presented modulation of TSPO function (reminiscent of the effect of TSPO knockdown, as mentioned) and presented the optimal concentration to a priori avoid non-effects of lower concentrations and to avoid confounding lethal effects of higher concentrations in this U118MG cell line. Furthermore, our first study to assay the effects of PK 11195 on gene expression in general showed a time dependent (from 24 to 48 h) effect of 25 µM of PK 11195 on gene expression [24], also reminiscent of the effects of TSPO knockdown on gene expression [19]. With the present study we wanted to apply shorter times of PK 11195 exposure to determine at which time points relevant changes in gene expression occur. We also wanted to optimize the application conditions for our gene expression studies. Therefore, we also took into consideration that full medium is known to be “activating” regarding gene expression [53,54]. Thus, full medium might interfere with the gene expression changes induced by PK 11195 or 2-Cl-MGV-1 and may complicate the issues at hand. This we strived to avoid. In this context, serum free culturing medium is considered to be optimal for gene expression studies as this renders cells quiescent [53,54]. Thus we applied serum free culturing medium. In short, we applied a straightforward paradigm and simple methods to clarify restricted questions.

We firstly found that changes in gene expression occur relatively early after PK 11195 application, i.e., already at 15 min. This is at least two hours before changes in physiological responses that are typically associated with TSPO function [40]. Secondly, we found that within 15 min the classical TSPO ligand PK 11195 induces considerable changes in gene expression associated with the canonical pathway for modulation of gene expression in general. We also found that not only the classical TSPO ligand PK 11195, but also the more advanced TSPO ligand 2-Cl-MGV-1 modulates expression of immediate early genes and transcription factors. These basic approaches indicate that one of TSPO’s functions is to modulate nuclear gene expression.

To further gain insights whether TSPO may modulate gene expression in a fairly direct way, we applied microscopic determination of the intracellular location of TSPO. A priori, we expected that after PK 11195 exposure TSPO may be found in the cell nucleus, as a result from various physiological changes. This then would allow for fairly direct control of nuclear gene expression. However, with our double labeling studies for TSPO, mitochondria, and cell nucleus, we always found TSPO to be located in the cytosol, including the mitochondria, and never in the nucleus of U118MG cells, with and without application of PK 11195, and at each time point of PK 11195 application, from 15 min till 24 h. The mitochondrial location of the TSPO suggested to us that the mitochondria-to nucleus signaling pathway is the main venue for regulation of gene expression by TSPO. This well-known mitochondria-to-nucleus communication pathway is conserved from yeast to humans and includes mitochondrial release of Ca^2+^, ATP, and ROS generation [27,28,29,30,31,32]. Interestingly, at all time points of PK 11195 exposure, we found that mitochondria intensely labeled for TSPO typically can be found relatively close to the cell nuclei. This never occurred with cells not exposed to TSPO. At 45 min of PK 11195 exposure, this was characterized by “caps” of mitochondrial populations expressing TSPO in the relative vicinity of the cell nuclei. We would like to believe that the relative close presence to the nucleus of mitochondria with TSPO may facilitate the regulation by TSPO of gene expression via mitochondria-to-nucleus signaling. Of course, alternative explanations are possible, such as enhanced energy requirements in particular subcellular regions [26]. In addition, it appears that within 15 min the application of PK 11195 causes the cells to contract to a round shape. This may contribute to the change of location of mitochondria toward the vicinity of the cell nucleus (see Figure 7). Or, the apparent location of the mitochondria is one of the contributing factors for cell shape change and motility. Then, already at 45 min the cells appeared to return from the morphology of clustered roundish cells back to polygonal cells evenly distributed over the culture plate. After 24 h, the cells have returned to their original appearances. Nonetheless, throughout this whole period of PK 11195 exposure, mitochondria with their TSPO remain visible close to the cell nucleus. Thus, we postulate that the morphological changes seen within 1 h of PK 11195 exposure, even starting already 15 min of PK 11195 exposure may contribute to, or at least be associated with, TSPO’s ability to induce early changes in gene expression. More studies, including high power light microscopy as well as electron microscopy, are needed to elucidate the microscopic observations of the present study. We are fully aware that a restricted number of other studies also show TSPO in other locations than mitochondria, including cell nuclei [7,46,47].

The mitochondrial location of the TSPO suggested to us that the mitochondria-to-nucleus signaling pathway is the main venue for regulation of gene expression by TSPO. As mentioned, this well-known mitochondria-to-nucleus communication pathway is conserved from yeast to humans and includes mitochondrial release of Ca^2+^, ATP, and ROS generation [27,28,29,30,31,32]. The mitochondria-to-nucleus signaling pathway, as outlined in the Introduction, includes mitochondrial ROS generation and loss of Δψm, leading to changes in levels of ATP and NADH, and the release of Ca^2+^, resulting in the activation of immediate early genes and transcription factors [29,30,31,32]. As also mentioned in the Introduction, TSPO regulates mitochondrial ROS generation, Δψm transitions, ATP production, Ca^2+^ release, and NADPH oxidase (NOX) activity [14,15,16,35,36,37,41,42,43,44]. In studies by others, effects on free radical generation by TSPO ligands have been studied in cultured neural cells, including primary cultures of rat brain astrocytes and neurons as well as cells of the murine BV-2 microglial cell line [55]. In these studies, free radical production was measured at the time points of 2, 30, 60, and 120 min of treatment with the TSPO ligands PK 11195, Ro5-4864, and PPIX (all at 10 nM). In astrocytes, all ligands showed a significant increase in free radical production at 2 min. Thus, ROS generation induced by classical TSPO ligands, synthetic and endogenous, apparently precedes changes in gene expression. As noted, such ROS generation, may be an essential component of the mitochondria-to-nucleus signaling for modulation of nuclear gene expression [26,56,57]. Finally, the present study shows that immediate early genes which are characteristic of the mitochondrial-to-nucleus pathway (e.g., *EGR1*, *FOS*, and *MYC*), are also induced by our application of PK 11195, the classical mitochondrial TSPO ligand. Thus it appears that the well reported primary location of the TSPO in the mitochondria [7,9,11], which was also observed in the present study, as well as TSPO’s well-known regulation of specific mitochondrial functions, favors a mitochondria-to-nucleus signaling pathway for TSPO’s ability to regulate gene expression. As strong quantitative changes in mitochondrial ROS generation, Δψm transitions, ATP production, Ca^2+^ release, and NADPH oxidase (NOX) activity typically are inductive for programmed cell death, we assume that moderate or small changes may rather be related to gene expression changes. This is a subject wanting for research.

We also wanted to see whether the changes in gene expression match generally known TSPO functions (as for example reviewed in [1,2,8,13]). To study this, we applied “Regulator Effects” analytic provided by Ingenuity (info-ingenuity@qiagen.com). Indeed, the functions derived from the gene expression changes matched with well-known TSPO functions. Briefly, “Regulator Effects” analytic indicates that PK 11195 exposure time-dependently induces functional changes related to: the cell cycle; programmed cell death; proliferation; migration; development, including cell differentiation; cell viability; inflammatory and immune responses; and tumorigenesis. In the present study, our microscopic observations also presented changes related to migration, development, and differentiation, even correlating in a timely fashion with the changes in expression of the relevant genes. Worldwide, careful TSPO research over the last 40 years has shown that PK 11195 and other TSPO ligands, as well as TSPO knockdown with genetic manipulation, modulate these same functions as seen with the gene expression analysis in this study [1,2,8,10,11,19,36,40,42,44,55,58,59,60].

Importantly, the pathway analysis showed that the gene expression changes presented interactive assemblies and super-assemblies. In short, in such assemblies, groups of genes provide several gene products for singular functions. In simple terms, the predictions of effects on specific functions appear to be robust. Moreover, the redundancies and degeneracies of pathways, forming the bases of the assemblies and super-assemblies, reinforce the robustness of functional gene expression effects induced by TSPO activity. It is well-known that redundancy and degeneracy in biological systems serve to stabilize them [49,50,51].

The experiments of the present study also provide data that functional changes predicted by observed changes in gene expression did actually occur. For example, the actual functional effects observed in the present study appear to include: stimulation of gene expression and accumulation of cells (at 15 min), activation of microtubule dynamics and cell motility (at 30 min), promotion of cell migration (at 45 min), then cell motility and accumulation of cells is reversed (at 3 h). Only at 1 and 24 h of PK 11195 expression no gene expression related to the actually observed functional effects was seen.

We were intrigued by the observed intracellular locality changes of TSPO labeling. It appears that because of some until now unknown cause and purpose, mitochondria in the relative vicinity of the cell nuclei enhance TSPO labeling in response to PK 11195 exposure. One alternative may be that mitochondria with TSPO can move from more distant areas in the cytosol to areas neighboring the cell nucleus, resulting in relative dense TSPO signal in such areas. This may potentially implicate that mitochondria not expressing TSPO are not motile in this paradigm. Such a phenomenon is not uncommon. For example in mature neurons, only one-third of axonal mitochondria are motile, the remainder thus being stationary [33]. Stationary mitochondria are considered to serve as local energy sources and buffer intracellular Ca^2+^ [33]. It is known that motility may serve to move mitochondria to the required intracellular locations for various cellular functions, such as proliferation, cell growth, cell cycle, differentiation, information transfer, apoptosis, etc. [31,56]. More studies are needed to determine whether TSPO actually may modulate mitochondrial motility.

Finally, as alluded above, also TSPO ligands other than PK 11195 can regulate gene expression. In the fifth approach of the present study this includes 2-Cl-MGV-1 which we found to modulate immediate early gene expression. As the affinity of 2-Cl-MGV-1 by design is relatively low, the concentrations of 2-Cl-MGV-1 given can be considered the equivalent of a 100 to 400 nM range of PK 11195. This is closer to the dissociation constant of PK 11195 than the 25 µM concentration used for PK 11195. Nota bene: in nature, a high affinity per se does not have to be advantageous. For example, the affinity of CO for hemoglobin is 210 higher than that of O_2_. However, CO is lethal, O_2_ is life giving. To further illustrate the potential general implications of our findings, other TSPO studies, applying genetic manipulation and ligands other than PK 11195 to target various specific functions, also showed modulations of gene expression [19,20,23,24,26,61,62,63].

**Figure 10:**
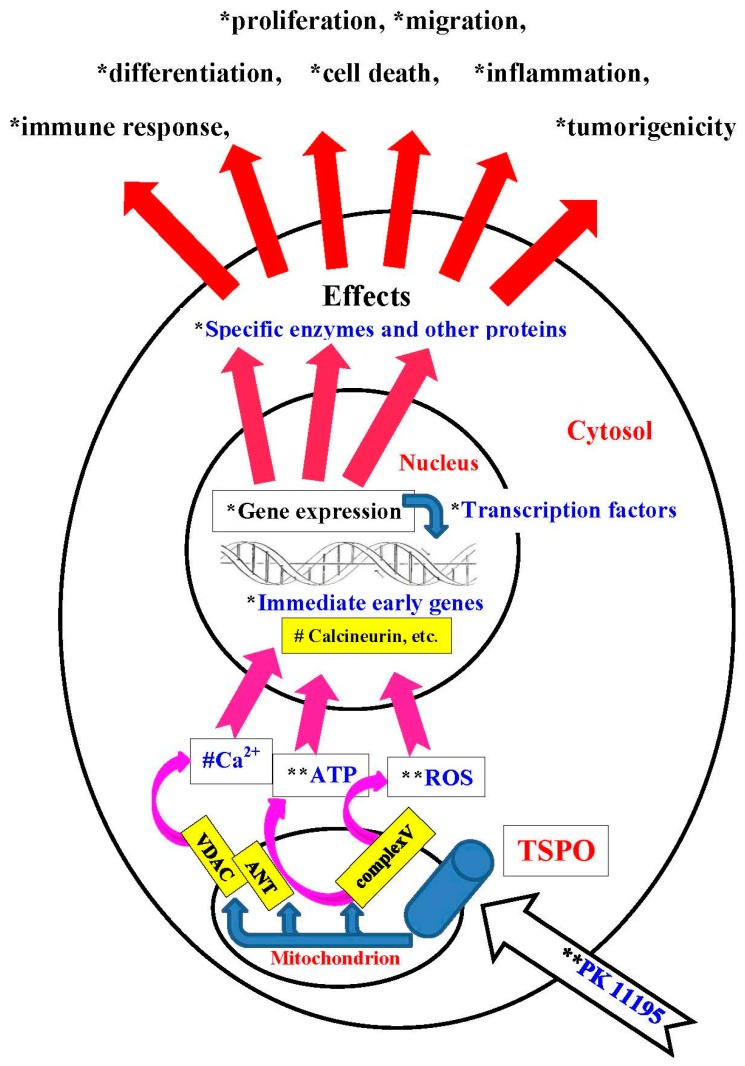
Regulation of gene expression by the TSPO ligand PK 11195. It is well known that the classical TSPO ligand PK 11195 can modulate mitochondrial TSPO functions such as Ca^2+^ release, ATP production, and ROS generation via modulation of mitochondrial proteins such as VDAC, ANT, and complexV (a.k.a. ATP(synth)ase). Ca^2+^ release, ATP production, and ROS are part of the mitochondrial – nuclear signaling pathway for regulation of nuclear gene expression. In this pathway, calcium sensitive proteins such as calcineurin and calmodulinKIV contribute to induction of expression of immediate early genes. The present study shows that PK 11195 exposure at first induces expression of immediate early genes, as well as other transcription factors (calcineurin, and calmodulinKIV are implicated in these effects), followed by changes in gene expression for enzymes and other proteins. Eventual potential functional implications include cell proliferation, cell migration, cell differentiation, cell death, inflammation, immune response, and tumorigenicity. While it is impossible to include all co-factors and context conditions into this diagram, it should be appreciated that by making small and big variations in the research paradigm, other final effects will become apparent regarding changes in function due to changes in gene expression, for example as a consequence of application of other TSPO ligands, TSPO knockdown, TSPO knockout, TSPO gene insertion, full medium, serum free medium, etc. The (*) and the (**) next to the phenomena modulated by PK 11195 indicate results from our present study (*) and from our previous studies (**). # indicates data from studies by others. (We consider it worthwhile for future studies to pay attention to the calcium sensitive proteins in the context of gene expression regulation via TSPO activities).

In summary, our study indicates that the TSPO ligand PK 11195 can modulate gene expression in U118MG cells within 15 min. This modulation involves regulation of expression of gene products that are part of the canonical pathway for regulation of gene expression. This gene expression appears to be related to cell viability and tumorigenicity of these U118MG cells. It is likely that such modulation in gene expression occurs via mitochondria-to-nucleus signaling, probably via mechanisms including ΔΨm collapse, ROS generation, Ca^2+^ release, and ATP production (Figure 10). It is well documented that these mechanisms are under the control of mitochondrial TSPO. The modulation of gene expression by the TSPO elucidated in the present study (Figure 10) goes a long way in explaining subsequent changes in cellular and organismal functions due to application of TSPO ligands. Thus, we propose that TSPO’s mitochondrial functions include modulation of nuclear gene expression via mitochondrial-nuclear signaling. This presents one way whereby TSPO can control several vital cell functions, which has major implications for the whole organism in health and disease.

### Caveats and Questions for Future Research

For most of its history, TSPO research has been challenged by oftentimes seemingly contradictory results. In this section here we only want to present a minimal sketch of this problem. For this short expose we provide a minimal number of references. For the interested reader, we refer to the numerous reviews regarding TSPO research, which directly or indirectly approach this enigma (not only referred to in this study, but also in other studies). It is well known that TSPO may contribute to various functions, sometimes appearing without any commonality [64,65,66,67,68,69]. One may find that for some researchers, gene regulation via TSPO or by TSPO ligands may be an undesired side effect. For others, it may be a desired feature, for example to render cancer cells less malignant, more differentiated, i.e., more like non-cancerous cells, or to induce regenerative responses to organ and tissue damage. These antagonistic expectancies regarding properties of TSPO and its ligands potentially may lead to widely divergent discrepancies in research approaches and interpretations of results. Another confounding aspect does relate to the now well appreciated concept that TSPO has homeostatic functions [20,23,26,38,62,63,70,71]. Assuming this to be true, many of the seemingly contradictory results can be explained as upregulatory and downregulatory characteristics of this potential homeostatic apparatus. An additional point, also redundancy and degeneracy potentially can make interpretations regarding TSPO properties difficult, as mechanisms affected by TSPO manipulations may be compensated for by other systems.It would be fascinating when somebody finds a hard-core, well defined, characteristic and/or mechanism whereby TSPO can exert its homeostatic functions.

A novel finding presented in Figure 8 should be discussed shortly. We observed two populations of mitochondria, one clearly expressing TSPO and one population of mitochondria showing no obvious presence of TSPO. To our knowledge, no quantitative studies have been performed regarding differential presence or no of TSPO in mitochondria of individual cells. Thus, our observation (striking to our minds) of mitochondria apparently with and without TSPO within one and the same cell may be a first. From another perspective, but potentially also interesting in this context, it has been observed that PK 11195 can regulate the conformation (folding or polymerization) of mitochondrial TSPO [72]. One can speculate that this regulation of TSPO protein structure may be one manner whereby immunological labeling of TSPO in mitochondria can be affected. At this point in time, one can only say that more studies are needed to resolve this matter.

Figure 10 in the manuscript simply presents the factual observations of the present study (indicated with * in Figure 10), as well as in previous studies by us (indicated with ** in Figure 10), and by others (indicated with # in Figure 10). Figure 10 also presents the core sequential associations between these observed facts. Table 6 below summarily lists these observations. Table 6 also points out potential caveats and presents questions that can be addressed by future research. Regarding each observation presented in the Table 6, it is obvious that a widely branched tree of assumptions, postulations, questions, and research approaches will emerge in relation to each observation, not to mention each potential caveat and questions associated with these factual observations. It is left to the TSPO enthusiast, and the general interested reader, which approaches based on their experience, expertise, and interests they feel should be pursued. It is obvious that with the very restricted number of TSPO studies available till now that the final word defining TSPO and its related mechanisms definitely has not been said. The authors here attempted to provide an aid for choice of questions for future research. It has been well recognized that TSPO presents a target that can be utilized for treatment of various diseases. In particular its characteristic as a receptor make it per definition a target for the development of ligands designed cure such diseases [22,45,71,73].

## 4. Experimental Section

### 4.1. Materials

Cell culture materials were obtained from Biological Industries (Beit Ha’emek, Israel). PK 11195 was from PerkinElmer (Hopkinton, MA, USA). Stock solution of PK 11195 was 10^−2^ M in 100% ethanol, kept at −20 °C. RNeasy Mini kit Qiagen and RNase–free DNase Set were from ELDAN (Petach Tikva, Israel). Human HT-12v4.0 Expression BeadChip Kit-24 samples were obtained via Danyel BIOTECH (Rehovot, Israel) from Illumina (San Diego, CA). DAPI Fluoromount-G^R^ was from Southern Biotech (Birmingham, AL, USA), MitoTracker^R^ Red CMXRos from Cell Signaling (Rehovot, Israel), anti TSPO primary antibodies from Abcam (Cambridge, UK), and secondary antibody (AffiniPure, Alexa fluor^R^ 488-conjugated, Donkey Anti Rabbit IgG(H + L)) from Jackson ImmunoResearch (Philadelphia, PA, USA).

#### Cell Culture

Cells of the human glioblastoma cell line U118MG were allowed to multiply under sterile conditions, at 37 °C, under humidified air with 5% CO_2_, in full medium, i.e., MEM-EAGLE supplemented with 10% fetal bovine serum (FBS), 2% glutamine, and 0.05% gentamycin, as described previously [2]. For the gene expression studies and the preferred approach of serum deprived medium was applied i.e., 0.5% FBS instead of 10% FBS, as this renders cells quiescent [53,54].

To study the effects of PK 11195 on neuronal differentiation, rat pheochromocytoma PC12 cells of flat, polygonal and attached phenotype, were generously provided by Ilana Gozes of the Tel Aviv University. This strain of PC12 cells has been studied extensively for differentiation effects of various types of TSPO ligands [20,22]. The PC12 cells were cultured in DMEM supplemented with 8% fetal calf serum (FCS), 8% heat-inactivated horse serum (HS), 1% glutamine and 0.1% penicillin (100,000 units/mL) + streptomycin (100 mg/mL) solution at 37 °C, 5% CO_2_, 90% relative humidity.

### 4.2. Exposure to PK 11195 and 2-Cl-MGV-1

U118MG cells (1.255 × 10^6^) were seeded in Petri dishes (i.e., 28.5 × 10^3^ cells/cm^2^) and allowed to proliferate for 3 days in full medium. Experiments for gene expression assayed with microarray typically consisted of three experimental groups and a vehicle control group (*n* = 3 for each group). Serum deprived medium was applied for 24 h, in the experimental groups ending with the inclusion of a choice from various TSPO ligand exposures. PK 11195 (25 µM final concentration) exposures were for 15, 30, 45 min, 1, 3, or 24 h (the vehicle control group is without PK 11195 inclusion). To verify whether other TSPO ligands could elicit gene expression effect, we also applied 25, 50, and 100 µM of 2-Cl-MGV-1 for one hour, according to the same paradigm as applied for PK 11195. 2-Cl-MGV-1 is a recently developed TSPO ligand with glial protective and neuronal differentiation effects [13,20,22,45].

Exposure to PK 11195 and 2-Cl-MGV-1 implies serum deprived medium with 1% alcohol (vehicle), PK 11195 (25 µM final concentration), or 2-Cl-MGV-1 (25, 50, and 100 µM) for the required time periods. Previously we had found that 25 µM of PK 11195 is the optimal concentration for various types of experiments with U118MG cells (e.g., [5,24,35,44]). The cells were collected by trypsinization, washed by centrifugation in phosphate buffered saline (PBS) (400× *g*, 5 min), and lysed (RLT lysis buffer provided with the RNeasy Mini Kit diluted with β-mercaptoethanol (1:100)), according to the manufacturer’s instructions. Lysates were stored at −70 °C.

### 4.3. RNA Extraction

Lysates from 3.85 × 10^6^ cells were taken for RNA extraction, according to the instructions of the RNeasy^®^ Mini Kit. Amount of obtained RNA was determined from a 1.5 µL sample by NanoDrop™ (Thermo, Rockland, DE, USA). For microarray assay, RNA was diluted to 50 ng/µL. RNA quality was verified in 1 µL of each sample [19,24].

### 4.4. Gene Expression Assay

100 ng of total RNA was amplified into biotinylated cRNA by in vitro transcription using the TargetAmp Nano labeling kit for Illumina BeadChips (Epicentre, an Illumina Company). The biotinylated cRNA was purified, fragmented, and hybridized to a HumanHT-12v 4.0 Expression BeadChip, according to the instructions of the Direct Hybridization assay (Illumina). The hybridized chip was stained with streptavidin-Cy3 (Amersham^TM^, GE Healthcare, Little Chalfont, UK) and scanned with an Illumina HiScan. The scanned images were imported into GenomeStudio (Illumina) for extraction and quality control. Then, the data were imported into JMP^®^ Genomics software version 6.0 (SAS Institute, Cary, NC, USA), and statistical analysis was applied. The raw data are at the NCBI data bank (File: GSE77998): http://www.ncbi.nlm.nih.gov/geo/query/acc.cgi?acc=GSE77998. And the second file (File: GSE85697) at: http://www.ncbi.nlm.nih.gov/geo/query/acc.cgi?acc=GSE85697.

### 4.5. Pathway Analysis

The raw data obtained with the microarray chip for gene expression assay were transformed to log2 and filtered out for transcripts with expression at background level. Background signal was provided by probes that contained scrambled DNA that does not correspond to any human DNA sequence. Transcripts were also filtered out when they only showed differences of <5% between the samples from the different time points (vehicle control = 0 h, and PK 11195 treatments for 15, 30, 45 min, 1, 3, and 24 h). Basic gene expression data analysis was performed using JMP^®^ Genomics version 6.0 (SAS, Cary, NC, USA). Principal component analysis and variance component analysis were performed and followed by one-way analysis of variance (ANOVA). For selection of individual genes with substantially changed expression, we applied a cut off of a minimal difference of 2-fold as well as an adjusted *p*-value of ≤0.05 for multiple comparisons [74]. Furthermore, Ingenuity Pathway Analysis (IPA^®^) software of QIAGEN [75] was applied. In particular, the “Regulator Effects” analytic (IPA^®^) was applied to obtain insights in the known downstream “Effects” (functional, phenotypic, disease related) related to genes with significant changes in expression (“Data Set”). Thus, the Data Set comprises genes with changed expression in our study. This software also shows the upstream “Regulators” (genes, RNAs, or proteins) that are known to be able to affect the genes of the “Data Set” in relation to their known functional, phenotypic, and disease related effects.

### 4.6. Real-Time RT-PCR

U118MG cells (1.255 × 10^6^) were seeded in Petri dishes (i.e., 28.5 × 10^3^ cells/cm^2^) for treatments, and allowed to proliferate for 3 days in full medium, just as for the microarray assay described above. Then, as described above, serum deprived medium was applied for 24 h, prior to PK 11195 exposures. We prepared for real-time reverse transcriptase (RT)-PCR according to procedures described previously [19,24]. Two biological duplicates were applied, each sample averaged from technical duplicates. Amount of obtained RNA was determined from a 1.5 µL sample by NanoDrop™ (Thermo, Rockland, DE, USA), as described above. RNA (1 µg) was reverse transcribed using the Verso^TM^ cDNA Synthesis Kit (Thermo Fisher Scientific, Waltham, MA, USA). The resulting cDNA was used as a template for TaqMan qPCR, using *DUSP*, *FOS*, and *B2M* specific primers and probes (Primer Design), and ABsolute^TM^ Blue qPCR ROX Mix (Thermo Fisher Scientific, Waltham, MA, USA). *B2M* was used as a normalizing gene. qPCR results were analyzed using Rotor-Gene Q 2.3.1.49 (QIAGEN) software.

### 4.7. Microscopic Studies

For triple labeling studies, cells were seeded in 6 well plates (each well holding a square microscopic cover glass) at the exact same cell density and growing conditions as applied for mRNA extraction i.e., 133 × 10^3^ cells per well. Stock solution of PK 11195 was diluted (to 25 × 10^−4^ M) in 100% ethanol. MitoTracker^R^ Red was dissolved in 100% DMSO (1 µM). For experiments, MitoTracker^R^ Red solution was diluted to a concentration of 100 nM in serum deprived medium (DMSO final concentration 0.01%). The treatments were 25 µM final concentration of PK 11195. Final concentration of the vehicle was 1% of ethanol.

The time periods of PK 11195 exposure for the microscopic studies were 0, 15, 30, and 45 min, and at 1, 3, and 24 h, ending together with the application of serum deprived medium for 24 h as described above. At the last hour of this 24 h period, MitoTracker^R^ Red of 100 nM was applied. Then the cells were fixated with 500 µL of 4% paraformaldehyde per well (2 × 20 min), and washed by PBS (2 × 5 min), followed by 0.5% Triton in PBS-500 µL per well for 10 min. Then blocking solution (5% bovine serum albumin (BSA) in PBS with 0.1% Triton) was applied for 1 h, followed by primary antibody against TSPO, at a final concentration of 1:100 in blocking solution TSPO (500 µL for each well), overnight at 4 °C. In one set, the primary antibody against TSPO was omitted. Next day, the cover glasses with the cells in the wells were washed with PBS (with 0.1% Triton, 2 × 5 min), and then the secondary antibody in blocking solution (1:100) for TSPO labeling was applied for 1 h at room temperature. Then the cover glasses with the cells were washed with PBS (2 × 5 min), and DDW (2 × 5 min), and coverslipped onto microscopic slides with DAPI Fluoromount-G^R^ and dried. Then these cells prepared for nuclear staining with DAPI, mitochondrial labeling with MitoTracker^R^, and TSPO immunofluorescent labeling with Alexa fluor^R^ 488, were observed with the aid of an Axio Observer z1 inverted microscope (Zeiss, Oberkochen, Germany), applying ZEN (Zeiss) for capture and image analysis.

For microscopic studies of neuronal differentiation induce by PK 11195, exposure for 48 h to 50 µM of PK 11195 was applied. Changes in cell morphology of differentiating PC12 cells include neurite sprouting, which is the hallmark of neuronal differentiation [20,22]. We used a Zeiss Axio observer inverted microscope, a Colibri led illumination light source, and a high resolution B/W CCD camera (Zeiss HS). Cell images were captured with the aid of Zeiss Axiovision 4.8 software for data acquisition.

### 4.8. Western Blot Analysis of Tubulin Expression in Relation to Neuronal Differentiation

Protein levels of cell pellets were measured according to Bradford [76] using bovine serum albumin as a standard. From PC12 cells treated with PK 11195 as described above, collected samples of with equal amounts of protein (20 µg protein/lane) were prepared in 1× sample buffer [0.125 M 2-amino-2-(hydroxymethyl) propane-1,3-diol (Tris)-HCl, pH 6.8, glycerol (20% *v*/*v*), SDS (sodium dodecyl sulfate) (0.1% *w*/*v*), 0.14 M β-mercaptoethanol, and bromophenol blue (0.005% *w*/*v*)]. The 12% SDS-polyacrylamide gels were run and analyzed as described previously [77]. To detect immunoreactivity related to our proteins of choice, we applied the appropriate primary antibodies: Monoclonal Anti-β-tubulin III (neuronal) Clone 2G10 from mouse (T8578, Sigma, Rehovot, Israel) at 0.5 µg/mL, as advised by the datasheet instructions. Monoclonal Anti-β-Actin antibody produced in mouse (A5441, Sigma, Rehovot, Israel) diluted 1:5000 was applied as a loading reference. The primary antibodies were labeled with IgG secondary antibody linked to horseradish peroxidase (anti-mouse IgG diluted 1:5000 as required; GE Healthcare, Buckinghamshire, UK). Binding of antibodies to their antigens was detected with the EZ-ECL-detection reagent. Labeling was captured on X-Omat blue XB-1 Kodak scientific Imaging Film. Band intensity was quantified by using Quantity one 1D-analysis software (Bio-Rad, Hercules, CA, USA).

## 5. Conclusions

- Our study indicates that the classical TSPO ligand PK 11195 can modulate gene expression in U118MG cells.
- Robust and significant changes in gene expression can already be seen within 15 min and appear to be associated with cell morphological changes within the same time frame.
- At least at 15 min of PK 11195 exposure, expression of several elements of the canonical pathway for regulation of gene expression in U118MG cells is enhanced.
- After 24 h of exposure to PK 11195, changes in gene expression appear to be related to cell viability and tumorigenicity of these U118MG cells.
- This modulation in gene expression most likely occurs via mitochondria-to-nucleus signaling, probably via mechanisms including ΔΨm collapse, ROS generation, Ca^2+^ release, and ATP production (Figure 9). It is well documented by previous studies that ΔΨm collapse, ROS generation, Ca^2+^ release, and ATP production are under the control of mitochondrial TSPO.
- Thus, TSPO does not just modulate local mitochondrial functions, it also modulates nuclear gene expression.
- Phenotypic changes predicted by the changes in gene expression did actually occur, e.g., cell migration, cell accumulation, cell differentiation, and others.
- The novel TSPO ligand 2-Cl-MGV-1 also specifically modulated gene expression of immediate early genes.
- The modulation of gene expression by the TSPO elucidated in the present study goes a long way in explaining subsequent changes in cellular and organismal functions due to application of TSPO ligands (Figure 9).
- Thus, modulation of nuclear gene expression via the mitochondrial TSPO can induce several vital cell functions, which has major implications for the whole organism in health and disease.
- We believe that our study provides more understanding in the overall biological function of TSPO.

## Figures and Tables

**Table 1:** Gene symbols of up-regulated genes and down-regulated genes with their expression changed due to exposure of U118MG cells to PK 11195 (25 µM) (in comparison to vehicle control in an exposure time related fashion) (cut off of 1.5): for 15, 30, 45, 60 min, 3, and 24 h.

**15–45 min of PK 11195 Exposure (25 µM)**					
15 min Up-regulated genes ↑		30 min Up-regulated genes ↑		45 min Up-regulated genes ↑	
*FOS*	↑ +2.16	*FOS*	↑ +3.96	*EGR1*	↑ +2.45
*DUSP1*	↑ +2.08	*DUSP1*	↑ +3.64	*PTGS2*	↑ +2.00
*EGR1*	↑ +1.89	*EGR1*	↑ +2.64	*FOS*	↑ +1.84
*CYR61*	↑ +1.82	*CYR61*	↑ +2.16	*DUSP1*	↑ +1.77
*OSR1*	↑ +1.71	*MYC*	↑ +2.03	*FOSB*	↑ +1.72
*ANP32AP1*	↑ +1.64	*CXCL8*	↑ +2.02	*CYR61*	↑ +1.69
*FKBP10*	↑ +1.54	*PTGS2*	↑ +2.01	*SGK1*	↑ +1.67
*RNA28S5*	↑ +1.54	*CTGF*	↑ +1.93	*CXCL8*	↑ +1.62
*DYNC1H1*	↑ +1.52	*NFKBIZ*	↑ +1.90	*ATF3*	↑ +1.58
*WNK1*	↑ +1.51	*SGK1*	↑ +1.89	*ANP32AP1*	↑ +1.58
15 min Down-regulated genes ↓		30 min Down-regulated genes ↓		45 min Down-regulated genes ↓	
*ID3*	↓ −1.83	*LOC100507412*	↓ −1.72	*MYLIP*	↓ −1.84
*TUFT1*	↓ −1.83	*TUFT1*	↓ −1.51	*LOC441087*	↓ −1.50
*ID2*	↓ −1.68	*MYLIP*	↓ −1.50		
*PTMA*	↓ −1.60	*KDM3A*	↓ −1.50		
*ID1*	↓ −1.56				
*KDM3A*	↓ −1.55				
*NABP1*	↓ −1.52				
*RPL21P28*	↓ −1.50				
*CLK1*	↓ −1.50				
**1–24 h of PK 11195 Exposure (25 µM)**					
1 h Up-regulated genes ↑		3 h Up-regulated genes ↑		24 h Up-regulated genes ↑	
*CYR61*	↑ +9.3	*ID3*	↑ +3.38	*ASN*	↑ +2.47
*FOSB*	↑ +4.29	*ID1*	↑ +2.08	*SLC7A5*	↑ +2.39
*EGR2*	↑ +3.32	*GBP1*	↑ +2.04	*TRIB3*	↑ +2.21
*EGR1*	↑ +3.2	*SMAD6*	↑ +1.89	*PCK2*	↑ +2.18
*CTGF*	↑ +3.04	*ID2*	↑ +1.86	*LOC729779*	↑ +2.12
*ID3*	↑ +2.62	*ATOH8*	↑ +1.85	*PSAT1*	↑ +1.97
*TUFT1*	↑ +2.49	*TXNIP*	↑ +1.84	*NUPR1*	↑ +1.94
*ID1*	↑ +2.33	*NEXN*	↑ +1.77	*P8*	↑ +1.88
*SRF*	↑ +2.15	*SLC3A2*	↑ +1.74	*DDIT4*	↑ +1.84
*GBP1*	↑ +2.12	*ACTG2*	↑ +1.71	*FAM102A*	↑ +1.82
*PTGS2*	↑ +2.11	*FHL2*	↑ +1.65	*SLC1A5*	↑ +1.8
*TRIB1*	↑ +2.09			*DDIT3*	↑ +1.8
*ERRFI1*	↑ +2.04			*ATF4*	↑ +1.77
*ATF3*	↑ +1.95			*TGIF1*	↑ +1.75
*KLF6*	↑ +1.74			*SPRR2D*	↑ +1.72
*FOS*	↑ +1.73			*PHGDH*	↑ +1.71
*DUSP5*	↑ +1.71			*SLC3A2*	↑ +1.69
*PTGER4*	↑ +1.69			*PLEKHF1*	↑ +1.67
*SGK*	↑ +1.69			*FOLR3*	↑ +1.65
*GADD45A*	↑ +1.69			*BEX2*	↑ +1.63
*SGK1*	↑ +1.69			*SLC6A15*	↑ +1.63
*ID2*	↑ +1.68			*IGFBP1*	↑ +1.56
*FILIP1L*	↑ +1.67				
1 h Down-regulated genes ↓		3 h Down-regulated genes ↓		24 h Down-regulated genes ↓	
*BCL6*	↓ −2.14	*IL8*	↓ −2.47	*MYLIP*	↓ −2.24
*DDIT4*	↓ −1.72	*MYLIP*	↓ −2.36	*UHRF1*	↓ −1.80
		*SOX4*	↓ −1.89	*IGFBP5*	↓ −1.79
				*RGS4*	↓ −1.72
				*PDE5A*	↓ −1.69
				*TYMS*	↓ −1.65
				*ERRFI1*	↓ −1.63

**Table 2:** Gene symbols of the genes with changed expression after15, 30, 45, 60 min, 3, and 24 h of PK 11195 exposure (of 25 µM), arranged according to their overall functions as analyzed with “Regulator Effects” analytic (IPA^®^). These overall functions are listed as: transcription factors, proteins, enzymes, and other products. Asterisks (*) indicate genes associated with programmed cell death. Other products include pseudogenes, ribosomal factors, etc. Further, the function of not all gene products is fully known. This time course presents 2 peaks for enhanced expression of transcription factors (at 15 min and at 1 h). Gene products including proteins, enzymes, and other products peak at 24 h.

Gene Expression Changes in U118MG Glioblastoma Cells after PK 11195 Exposure for different Time Periods					
15 min	30 min	45 min	60 min	3 h	24 h
All Types of Genes Combined					
20 Genes	14 Genes	12 Genes	25 Genes	14 Genes	29 Genes
**Transcription Factors**					
			*ATF3* *		
			*BCL6* *		
			*DUSP5*		
*CLK1*			*EGR1* *		
*DUSP1* *			*EGR2* *		
*EGR1* *			*FOS* *		
*FOS* *			*FOSB* *		
*OSR1* *			*GADD45A* *		
*ID1* *	*DUSP1* *		*ID1* *	*ATOH8*	*ATF4* *
*ID2* *	*EGR1* *	*ATF3* *	*ID2* *	*ID1* *	*DDIT3* *
*ID3* *	*FOS* *	*DUSP1* *	*ID3* *	*ID2* *	*NUPR1* *
*KDM3A* *	*KDM3A*	*EGR1* *	*KLF6* *	*ID3* *	*TGIF1*
*NABP1*	*MYC* *	*FOS* *	*SRF*	*SOX4*	*TRIB3* *
*PTMA* *	*NFKBIZ* *	*FOSB* *	*TRIB1* *	*SMAD6* *	*UHRF1*
(11 genes)	(6 genes)	(5 genes)	(14 genes)	(6 genes)	(6 genes)
**Proteins, Enzymes, and other Products**					
					*ASNS* *
					*BEX2* *
					*DDIT4* *
					*ERRFI1*
					*FAM102A*
					*FOLR3*
					*IGFBP1* *
					*IGFBP5* *
					*LOC729779*
					*MYLIP*
					*PCK2*
					*PDE5A*
			*CTGF* *		*PHGDH*
			*CYR61* *		*PLEKHF1*
*ANP32AP1*			*DDIT4* *		*P8*
*CYR61* *	*CTGF* *		*ERRFI1*	*ACTG*	*PSAT1*
*DYNC1H1* *	*CXCL8*	*ANP32AP1*	*GBP1*	*FHL2* *	*RGS4*
*FKBP10*	*CYR61* *	*CXCL8*	*FILIP1L*	*GBP1*	*SLC1A5*
*MIR22HG*	*LOC100507412*	*CYR61* *	*PTGER4*	*IL8*	*SLC3A2*
*RNA28S5*	*MYLIP*	*LOC441087*	*PTGS2* *	*MYLIP*	*SLC6A15*
*RPL21P28*	*PTGS2* *	*MYLIP*	*SGK*	*NEXN*	*SLC7A5*
*TUFT1*	*SGK1* *	*PTGS2* *	*SGK1* *	*SLC3A2*	*SPRR2D*
*WNK1*	*TUFT1*	*SGK1* *	*TUFT1*	*TXNIP* *	*TYMS*
(9 genes)	(8 genes)	(7 genes)	(11 genes)	(8 genes)	(23 genes)

**Table 3:** Real-time RT-PCR analysis (*C*_t_) of *FOS*, *DUSP1*, and *B2M* expression in glioblastoma cells after 30 min exposure to 25 µM of PK 11195. The presentation of the *C*_t_ data as means ± SD. *** *p* < 0.001, ** *p* < 0.01, *n* = 2 (One way ANOVA, posthoc Bonferroni, multiple comparisons) shows the statistical significances of the differences between vehicle (i.e., untreated control) and PK 11195 (i.e., the treated groups) for *FOS* and *DUSP.* Each member of the biological duplicates is the average of 2 technical duplicates. Biological duplicates means cells grown in 2 different wells i.e., truly independent measurements. Technical duplicates means two measurements on the same biological sample (to achieve better accuracy); their average in the end thus is just one measurement.

*C*_t_	Vehicle	PK 11195
B2M	22.10 ± 0.85	21.85 ± 0.21 n.s.
FOS	29.35 ± 0.07	26.20 ± 0.28 ***
DUSP1	24.90 ± 0.42	22.85 ± 0.21 **

**Table 4:** Functional effects implied by the gene expression modulated by PK 11195. This was acquired by application of “Regulator Effects” analytic (IPA^®^). The time points of 15, 30, 45 min, 1, 3, and 24 h applied in this study are given, providing a time-course of functional changes. (The modulated gene expression itself is presented in Table 1 and Table 2.) In the appendices more detailed presentations of the outcomes of “Regulator Effects” analytic (IPA^®^) are given for each time point. Functions that are upregulated are given here in bold red, functions that are down regulated are given in blue.

15 min	30 min	45 min	1 h	3 h	24 h
Gene expression modulation Binding of protein binding site Transactivation of RNA Endothelial cell development Cell viability Accumulation of cells	Binding of protein binding site Synthesis of DNA Differentiation of connective tissue cells Development of neurons Formation of cells Microtubule dynamics Chemotaxis of cells Cell movement of fibroblast cell lines Metastasis of tumor cell lines Abdominal neoplasm Proliferation of lymphocytes Growth of tumor Cell viability Metabolism of carbohydrate Inflammation of body region	Synthesis of DNA Development of neurons Formation of cellular protrusions Angiogenesis Proliferation Migration Cell growth	Apoptosis of fibroblast cell lines Malignant solid tumor S phase Cell cycle progression of fibroblast cell lines Development of cardiovascular system Cell viability Formation of cellular protrusions Growth of malignant tumor Proliferation of tumor cells Formation of cells Development of reproductive system	Cell death of central nervous system cells Apoptosis of fibroblasts Apoptosis of myeloid cells Apoptosis of muscle cell lines Necrosis of epithelial tissue Migration of colon cancer cell lines Cell movement of leukocyte cell lines Migration of smooth muscle cells Migration of phagocytes Chemotaxis Cell viability Development of epithelial tissue Proliferation of leukocyte cell lines Activation of leukocytes Inflammatory response Accumulation of leukocytes Proliferation of leukemia cell lines Activation of tumor cell lines	Cell death of fibroblast cell lines Apoptosis of kidney cell lines Apoptosis of epithelial cell lines Abdominal cancer Digestive system cancer Growth of digestive organ tumor Growth of malignant tumor Epithelial cancer Proliferation of tumor cells

**Table 5:** Dose dependent changes in gene expression of U118MG cells due to exposure 2-Cl-MGV-1.

50 µM		100 µM	
Up-regulated genes ↑		Up-regulated genes ↑	
*FOS*	↑ 2.5	*FOS*	↑ 3.46
*ZFP36*	↑ 1.68	*ZFP36*	↑ 1.72
*DUSP1*	↑ 1.62	*DUSP1*	↑ 1.71
Down-regulated genes ↓		Down-regulated genes ↓	
*TUFT1*	↓ 1.64	*ID2*	↓ 1.68

**Table 6:** Caveats and future questions regarding this and other TSPO research.

Observations	Caveats or Questions	Answer or Future Studies
PK 11195 applications affect gene expression and the related functions.	Is this context dependent?	The application of PK 11195 can be further refined (dose, time window, co-factors, etc.).
TSPO ligands other than PK 11195 affect gene expression and related functions.	Is this context dependent?	If desired, the application of ligands other than PK 11195 can be further refined (dose, time window, co-factors, etc.).
TSPO knockdown affects gene expression and related functions.	Is this context dependent?	If desired, the application of TSPO knockdown and knockout can be further refined (transient, stable, time course of effects, co-factors, etc.).
Interaction between PK 11195 and TSPO. PK 11195 can interact with TSPO to finally affect gene expression.	PK 11195 can also bind to other receptors. This can also occur via other receptors.	Knockout all the other receptors binding to PK 11195 (Difficult in practice). TSPO can be knocked down to address this question (This has been done). It can be checked whether other TSPO ligands can affect gene expression (This has been done).
Interactions between TSPO and PK 11195 affect mitochondrial to nuclear signaling, for example via the initiating steps of ROS regeneration, and Ca^2+^ and ATP release.	Will different concentrations of ATP and Ca^2+^ and levels of ROS relate to different changes in gene expression?	ATP and Ca^2+^ levels and ROS generation can be measured after TSPO manipulations. Gene expression can be measured. Protein expression of calcium sensitive proteins can be measured.
Mitochondrial to nuclear signaling apparently induced by TSPO and its ligands implicates various calcium sensitive proteins.	Which calcium sensitive proteins can be activated due to TSPO modulations?	One can measure calcium binding proteins after TSPO manipulations.
Immediate early genes and other transcription factors are activated as a consequence of TSPO knockdown and TSPO ligand applications.	TSPO ligands can act via other receptors than TSPO. TSPO knockdown can possibly be compensated by various cellular mechanisms.	One should not rely on one method to induce effects of TSPO manipulation on gene expression. The time interval between measurements after TSPO manipulation, such as knockdown or knockout, or application of TSPO ligands, or other agents affecting TSPO activity should be as short as possible. In this way, compensatory events are precluded.
Immediate early genes induce changes in expression of other genes.	How is it ‘decided’ which genes will be modulated? One molecule such as PK 11195 by itself cannot determine which complex of genes will change expression.	TSPO modulation has to be combined with co-factors or different contexts. The assumption is that these additional variations, together with TSPO modulation determine the patterns in changes of gene expression. For example switching from minimal to maximal cell culture medium results in major changes in numbers of gene expression changes.
The final changes in gene expression correlate with functional changes.	Does this always occur?	Modulation of TSPO activity, modulation of gene expression, and modulation of function always have to be considered in association with each other and subjected to combined studies (an approach taken in the present study).
Modulation of gene expression typically has functional effects.	How important is control of TSPO and its ligands of gene expression?	We think it is very important, at least in cell culture and also in animal models we have seen major phenotypic changes.

## Supplementary Materials

Supplementary materials can be found at www.mdpi.com/1422-0067/18/4/786/s1.
