# Supplementary material for: Classical and Novel TSPO Ligands for the Mitochondrial TSPO Can Modulate Nuclear Gene Expression: Implications for Mitochondrial Retrograde Signaling
Source: Int J Mol Sci. 2017 Apr 7;18(4):786. doi: 10.3390/ijms18040786 (PMC5412370; doi:10.3390/ijms18040786)
Supplement: Supplementary file 1 [file ijms-18-00786-s001.pdf]

[IJMS] Manuscript ID: ijms-131864 –

Supplementary Materials: Figures and Tables accompanying the manuscript :

Classical and novel TSPO ligands for the mitochondrial TSPO can modulate nuclear gene expression: Implications for mitochondrial retrograde signaling.

Yasin N†, Veenman L†, Maya Ezrad, Caballero B, Gavish M*

This supplementary files provide the comprehensive details of the pathway analysis with Ingenuity IPA® for all time points assayed (15, 30, 45 minutes, 1, 3, and 24 hours), including presentations of 'Regulators', 'Data Sets', and 'Effects' . Note: the 'Data Sets' are genes with changed gene expression that are associated with 'Regulators' that via the genes in question are known to exert particular 'Effects' . The 'Effects' pertain to functions, phenotypes, and diseases.

The images are supported by lists and tables, clarifying the acronyms, and gene symbols.

Also the canonical pathways for functional implications of changes in gene expression due to the PK 11195 (25 μM) applications of the present study are given for 15, 30, and 45 minutes of PK 11195 exposure.

Supplementary file 1 : 15 minutes of exposure to 25 µM of PK 11195 of U118MG cells (canonical pathway of figure 1 in the manuscript indicating that within 15 minutes of exposure to 25 µM of PK 11195 genes are upregulated for components that are part of the canonical pathway for regulation of gene expression).

The genes in question are marked in red and light red.

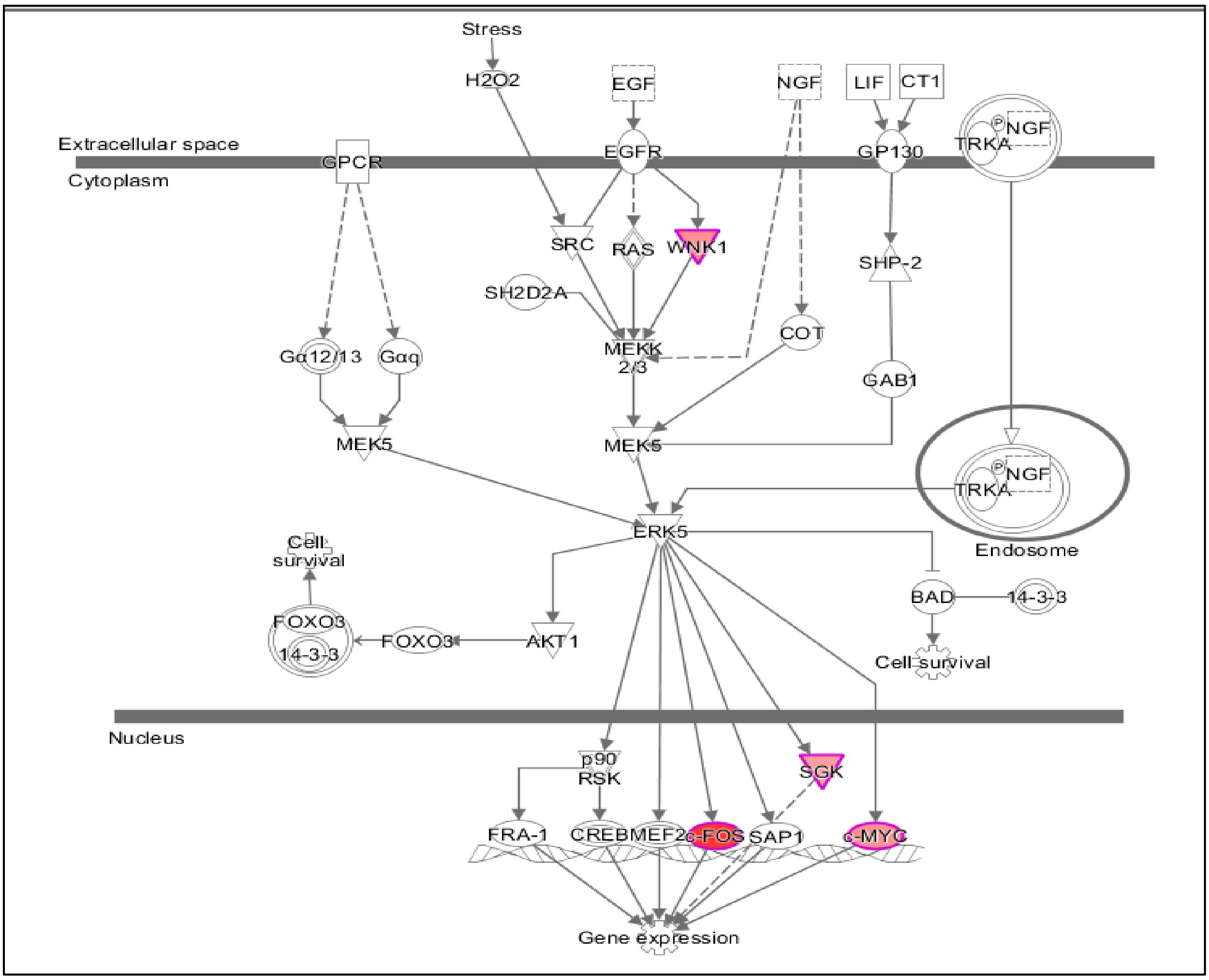

Supplementary file 2 : 15 minutes of exposure to 25 µM of PK 11195 of U118MG cells (Figure 2 in ms.)

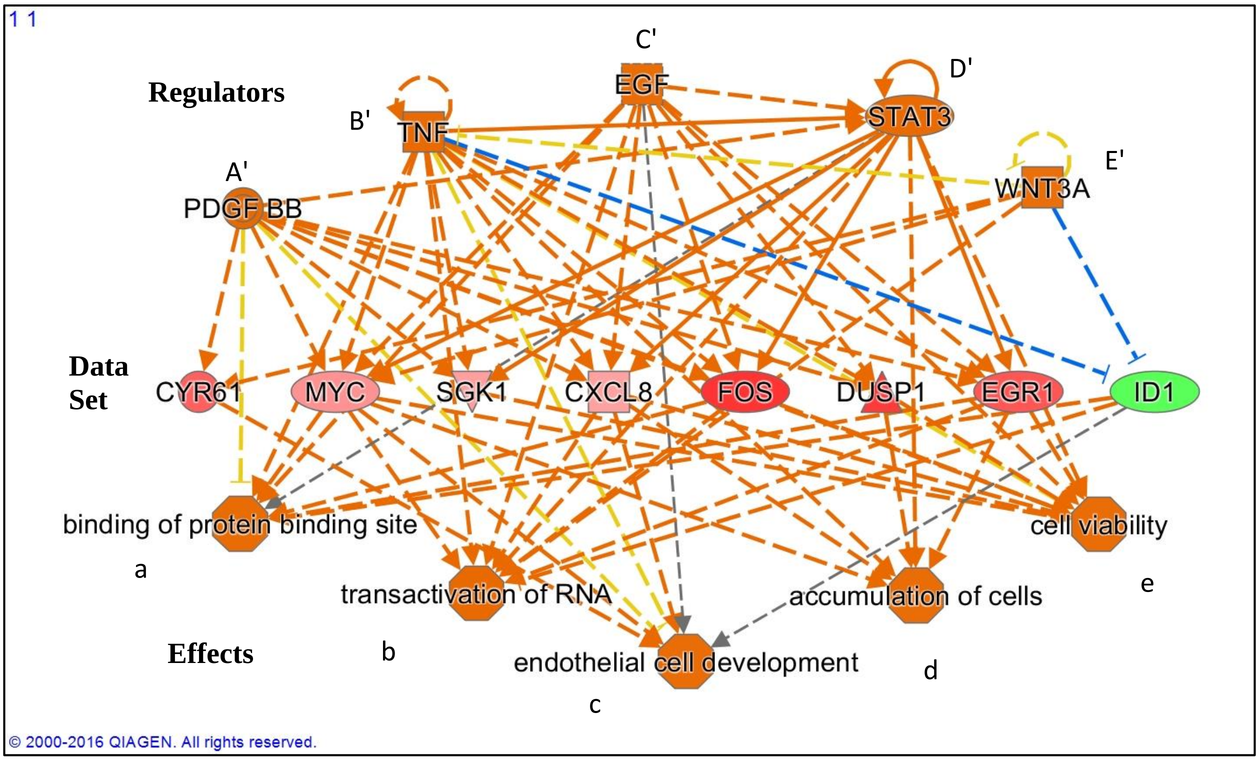

Potential effects on cellular functions due to the significant changes in gene expression as induced by 15 minutes of exposure of U118MG cells to PK 11195 (25 µM). The pathway analysis (Regulator Effects analytic IPA® applying adjusted p ≤ 0.05) indicates multiple interactive upstream pathways ('Regulators' given in the upper tier) related to the genes with significantly changed expression ('Data Set' given in the middle tier), together with the various downstream functions ('Effects' in the bottom tier). This whole configuration can be annotated as a super-assembly (see text for more detailed explanation). Color coding: In the 'Data Set' (middle tier): pink/red = upregulated, green = down regulated. In the 'Effects' (bottom tier) and in the 'Regulators' (top tier) the predictions are indicated by orange = upregulated.

Supplementary file 3 : 15 minutes of exposure of U118MG cells to PK 11195 (25 µM). This is the list of 'Regulators' of the figure of file 2, detailing their known modulation of the genes of the 'Data Set' and the consequential 'Effects'.

This Table corresponds to Table 1 in the manuscript

	Upstream Regulator	Molecule Type	Target genes in dataset with changed expression p < 0.5	Effects
**A'**	**PDGF BB**	Complex (Extracellular Space)	**CYR61↑, MYC↑, SGK1↑, CXCL8↑, FOS↑, DUSP1↑, EGR1↑**	a,c,e
**B'**	**TNF**	tumor necrosis factor	**MYC↑, SGK1↑, CXCL8↑, FOS↑, DUSP1↑, EGR1↑,** ID1 ↓	a,b,c,d,e
**C'**	**EGF**	epidermal growth factor	**MYC↑, CXCL8↑, FOS↑, DUSP1↑, EGR1↑**	a,b,c,e
**D'**	**STAT3**	signal transducer and activator of transcription 3	**MYC↑, SGK1↑, CXCL8↑, FOS↑, EGR1↑**	a,b,d,e
**E'**	**WNT3A**	Cytokin Wnt family member 3A	**CYR61↑, MYC↑,** ID1↓	b

Table Supplementary file 3. Details regarding the 'Regulators' of **Figure in supplementary file 2.** The list of primed capital lettering (first column) matches the 'Regulators' marked with the same lettering in the upper tier of Figure 2. The acronyms of the 'Regulators are given (second column). The molecular type of the 'Regulators' is summarized (third column). The gene symbols of the target genes of each of the 'Regulators' are listed (fourth column). Upregulation of gene expression is indicated in **'bold font'** and the corresponding arrows. Downregulation is indicated in 'grey font' and downward pointing arrows. The final 'Effects' (fifth column) induced by the 'Regulators' and their targeted genes, are indicated with lower case lettering in the fifth column, according to the same lettering in Figure 2 marking the 'Effects' in the lower tier. Thus each Regulator affects a group of genes associated with a group of functions. Furthermore, these groups of genes and functions overlap. This configuration is defined as a super-assembly (see text for more detailed explanations).

Supplementary file 4 : 30 minutes of exposure to 25 µM of PK 11195 of U118MG cells

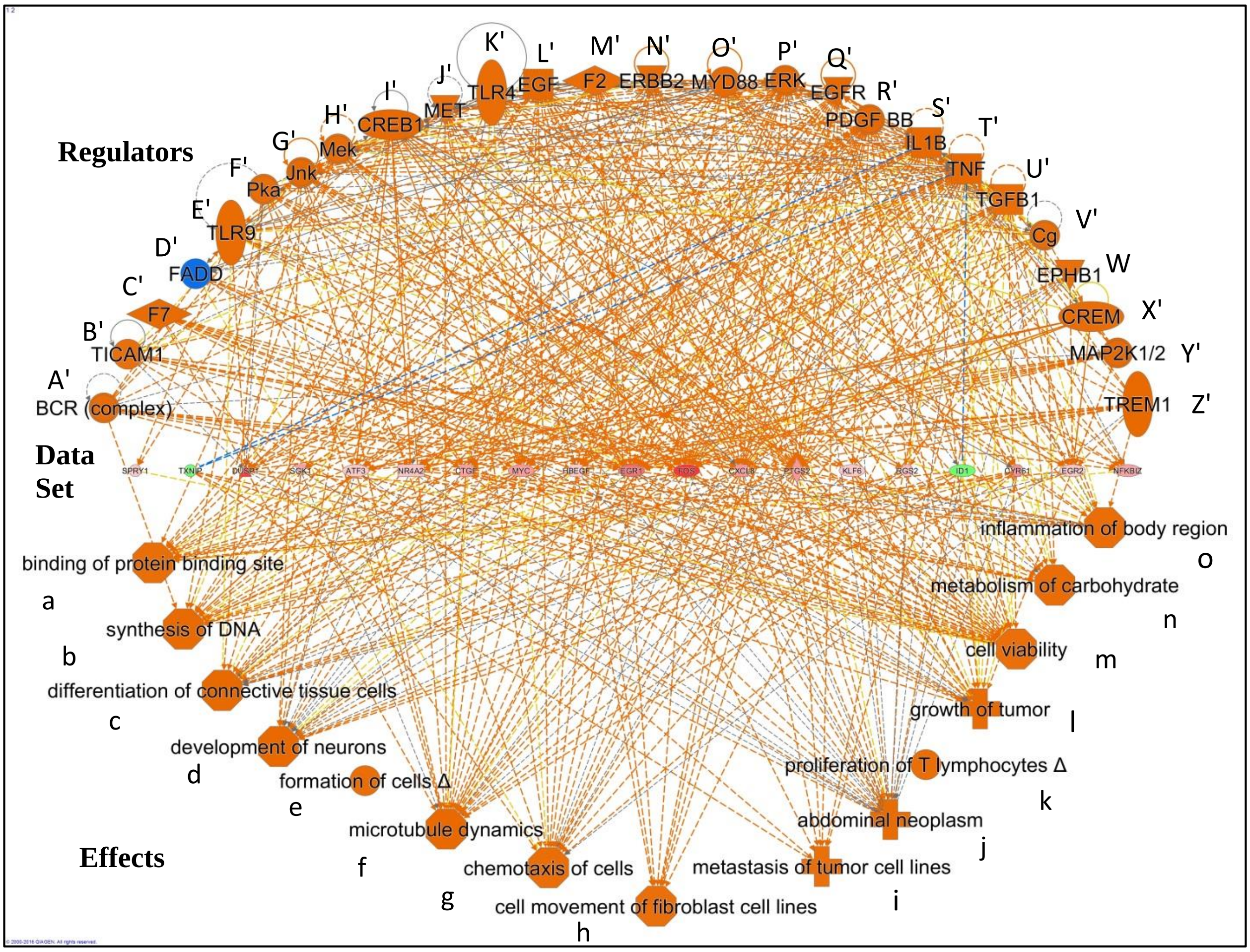

The Genes of the 'Data Set' (middle tier) from left to right : SPRY1, TXNIP, DUSP1, SGK1, ATF3,NR4A2, CTGF, MYC, HBEGF, EGR1, FOS, CXCL8, PTGS2, KLF6, RGS2, ID1, CYR61, EGR2, NFKBIZ

Potential effects on cellular and tissue functions due to the significant changes in gene expression as induced by 30 minutes of exposure of U118MG cells to PK 11195 (25 µM). The pathway analysis (Regulator Effects analytic IPA® applying adjusted $p \leq 0.05$) indicates multiple interactive upstream pathways ('Regulators' given in the upper tier) related to the genes with significantly changed expression ('Data Set' given in the middle tier), together with the various downstream functions ('Effects' in the bottom tier). This whole configuration can be annotated as a super-assembly (see text for more detailed explanations). Color coding: In the 'Data Set' (middle tier): pink/red = upregulated, green = down regulated. In the 'Effects' (bottom tier) and in the 'Regulators' (top tier) the predictions are indicated by blue = downregulated, and orange = upregulated. The row of gene symbols at the bottom of the figure are those of the middle tier ('Data Set') of this figure presented in the same sequence from left to right. The color coding indicates upregulation (red) and downregulation (blue).

Supplementary file 5 : 30 minutes of exposure to 25 µM of PK 11195 of U118MG cells. List of 'Regulators' of File 4, detailing their known modulation of the genes of the 'Data Set' and the consequential 'Effects'.

	**Upstream Regulator**	**Molecule Type**	**Target genes in dataset with changed expression P<0.5**	**Effects**
**A'**	BCR (complex)	complex	**MYC↑, EGR1↑, FOS↑, PTGS2↑**	b
**B'**	TICAM1	toll like receptor adaptor molecule 1	**DUSP1↑, EGR1↑, CXCL8↑, NFKBIZ↑**	j,m,o
**C'**	F7	coagulation factor VII	**CTGF↑, MYC↑, HBEGF↑, EGR1↑, FOS↑,**	f,g,m
**D'**	FADD	Fas associated via death domain	**MYC↑, EGR1↑, FOS↑, KLF6↑**	g,l,m
**E'**	TLR9	toll like receptor 9	**SPRY1↑, DUSP1↑, ATF3↑, EGR1↑, CXCL8↑, NFKBIZ↑**	c,m
**F'**	Pka	complex, enzyme, kinase	**NR4A2 ↑, CTGF↑, EGR1↑, FOS↑, RGS2↑**	b,c,f,m,n
**G'**	Jnk	Jnk dimer	**CTGF↑, FOS↑, CXCL8↑, KLF6↑**	d,f,g,m,n
**H'**	Mek	Erk Kinase, alcohol group acceptor phosphotransferase	**SPRY1↑, MYC↑, CXCL8↑, PTGS2↑**	a,b,j,l,m
**I'**	CREB1	cAMP responsive element binding protein 1	**ATF3↑, NR4A2↑, MYC↑, EGR1↑, FOS↑, CXCL8↑, PTGS2↑, RGS2↑, CYR61↑, EGR2↑**	b,c,d,f
**J'**	MET	Met dimer	**MYC↑, HBEGF↑, NFKBIZ↑**	d,f,i,j,l,m
**K'**	TLR4	toll like receptor 4	**ATF3↑, NR4A2↑, HBEGF↑, CXCL8↑, PTGS2↑, CYR61↑, NFKBIZ↑**	c,f,g,j,l,m,o
**L'**	EGF	EGFR ligand	**DUSP1↑, MYC↑, EGR1↑, FOS↑, CXCL8↑, PTGS2↑, EGR2↑**	a,b,f,g,h,j,l, m,n
**M'**	F2	coagulation factor II, thrombin	**CTGF↑, EGR1↑, FOS↑, CXCL8↑, PTGS2↑**	a,b,c,f,h,j,l, m,n,o
**N'**	ERBB2	erb-b2 receptor tyrosine kinase 2	**CTGF↑, MYC↑, HBEGF↑, FOS↑, CXCL8↑, PTGS2↑**	a,b,d,f,g,j,l, m
**O'**	MYD88	myeloid differentiation primary response 88	**DUSP1↑, EGR1↑, CXCL8↑, PTGS2↑, CYR61↑, NFKBIZ↑**	c,d,f,g,j,l,m, n,o
**P'**	ERK	[RNA-polymerase]-subunit kinase	**DUSP1↑, CTGF↑, HBEGF↑, EGR1↑, FOS↑, CXCL8↑, PTGS2↑, EGR2↑, NFKBIZ↑**	a,b,c,d,f,g,j, m,o
**Q'**	EGFR	epidermal growth factor receptor (actin filament binding)	**CTGF↑, MYC↑, HBEGF↑, EGR1↑, FOS↑, CXCL8↑**	a,b,c,d,f,g,h, i,j,l,m
**R'**	PDGF BB	Pdgf (complex)	**DUSP1↑,SGK1↑,ATF3↑,NR4A2↑, CTGF↑, MYC↑, HBEGF↑, EGR1↑, FOS↑, CXCL8↑, PTGS2↑, KLF6↑, RGS2↑**	a,b,c,d,f,g,h, m,n,o
**S'**	IL1B	interleukin 1 beta	TXNIP↓, **DUSP1↑,ATF3↑ NR4A2↑, EGR1↑, FOS↑, CXCL8↑, PTGS2↑, NFKBIZ↑**	a,b,c,d,f,g, j,l,m,n,o
**T'**	TNF	tumor necrosis factor (cleavage site, cytokine, identical protein binding)	TXNIP↓, **DUSP1↑, SGK1↑,ATF3↑,NR4A2↑, MYC↑, HBEGF↑, EGR1↑, FOS↑, CXCL8↑, PTGS2↑, KLF6↑, RGS2↑,** ID1↓, **NFKBIZ↑**	a,b,c,d,f,g,i,j , l,m,n,o
**U'**	TGFB1	transforming growth factor beta 1	**CTGF↑, MYC↑, HBEGF↑, FOS↑, CXCL8↑, PTGS2↑** ID1↓, **EGR2↑**	a,b,c,d,f,g,h, i,j,l,m,n,o
**V'**	Cg	complex	**DUSP1↑, EGR1↑, CXCL8↑, PTGS2↑, RGS2↑**	a,b,j,n
**W'**	EPHB1	EPH receptor B1	**EGR1↑**	d,f,g
**X'**	CREM	cAMP responsive element modulator	**FOS↑, PTGS2↑, EGR2↑**	b,l
**Y'**	MAP2K1/2	MEK1/2, MKK1/2	**ATF3↑,NR4A2↑, EGR1↑, FOS↑**	a,l
**Z'**	TREM1	triggering receptor expressed on myeloid cells 1	**DUSP1↑ ,ATF3↑, CTGF↑, MYC↑, EGR1↑, EGR2↑**	g,o

Table Supplementary file 5. Details regarding the 'Regulators' of the figure of File 4. The list of primed capital lettering (first column) matches the 'Regulators' marked with the same lettering in the upper tier of Figure 3. The acronyms of the 'Regulators are given (second column). The molecular type of the 'Regulators' is summarized (third column). The target genes of each of the 'Regulators' are listed (fourth column). Upregulation of gene expression is indicated in **'bold font'** and the corresponding arrows. Downregulation is indicated in 'grey font' and downward pointing arrows. The final 'Effects' (fifth column) induced by the 'Regulators' and their targeted genes, are indicated with lower case lettering in the fifth column, according to the same lettering in Figure 3 marking the 'Effects' in the lower tier. Thus each Regulator affects a group of genes associated with a group of functions. Furthermore, these groups of genes and functions overlap. This configuration is defined as a super-assembly (see text for more detailed explanations).

Supplementary file 6 : 30 minutes of exposure of U118MG cells to PK 11195 (25 µM).

(canonical pathway as provided by pathway analysis with Ingenuity IPA® showing that within 30 minutes of exposure to 25 µM of PK 11195 genes are upregulated for components that are part of the canonical pathway for regulation of angiogenesis and tumor growth).

The genes in question are marked in red and light red.

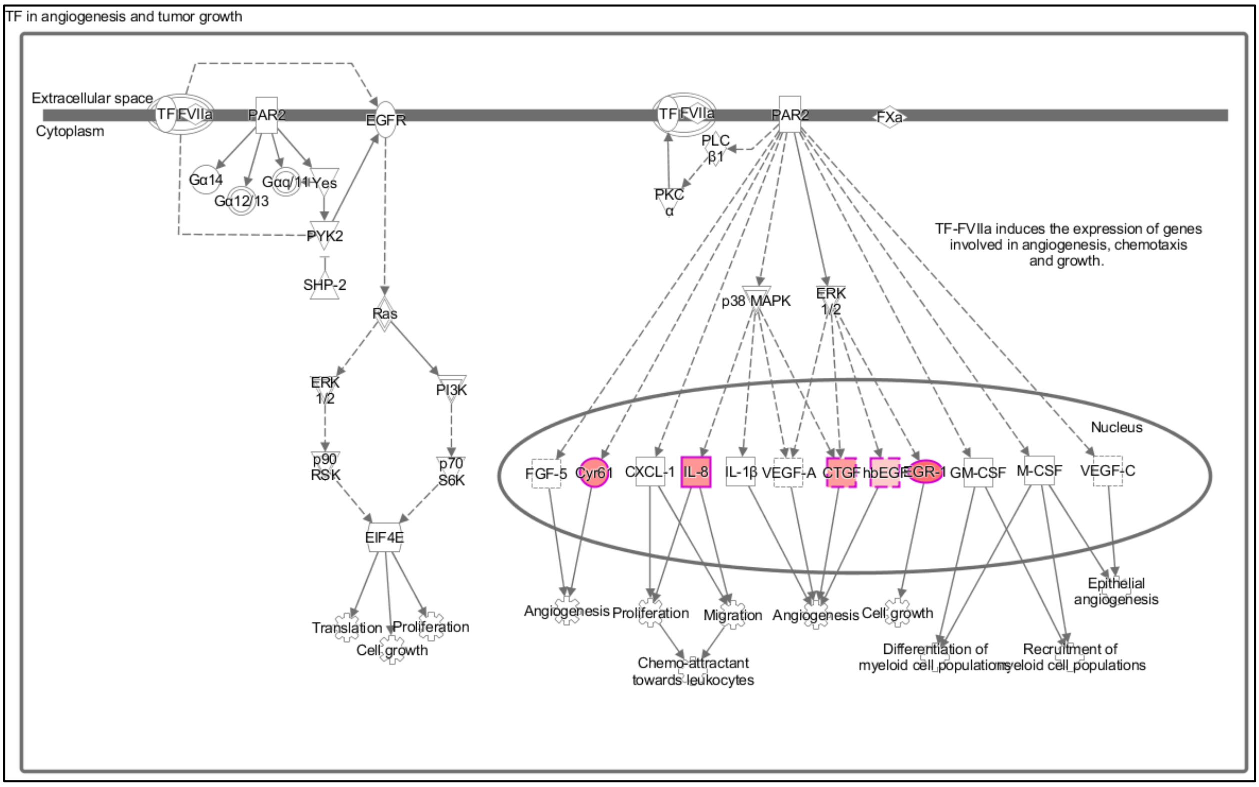

Supplementary file 7 : 45 minutes of exposure to 25 µM of PK 11195 of U118MG cells

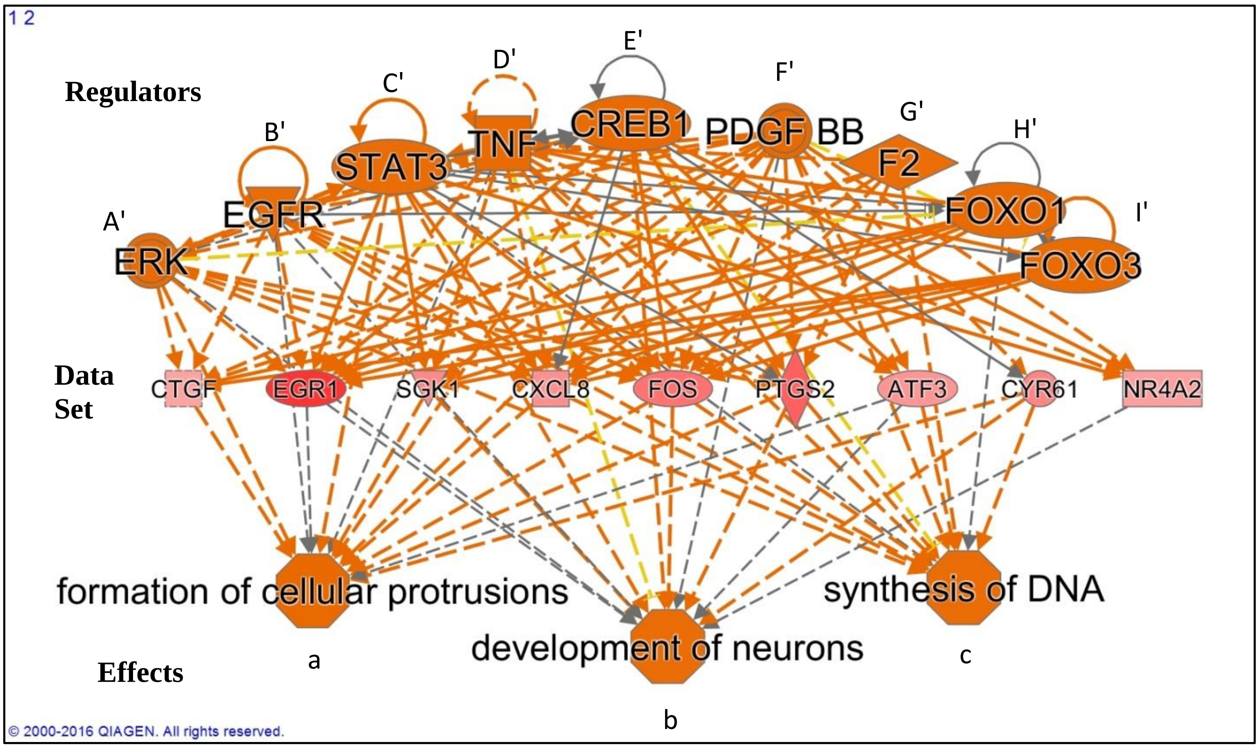

Potential effects on cellular functions due to the significant changes in gene expression as induced by 45 minutes of exposure of U118MG cells to PK 11195 (25 µM). The pathway analysis (Regulator Effects analytic IPA® applying adjusted $p \leq 0.05$) indicates multiple interactive upstream pathways ('Regulators' given in the upper tier) related to the genes with significantly changed expression ('Data Set' given in the middle tier), together with the various downstream functions ('Effects' in the bottom tier). This whole configuration can be annotated as a super-assembly (for detailed explanation, see text). Color coding: In the 'Data Set' (middle tier): pink/red = upregulated. In the 'Effects' (bottom tier) and in the 'Regulators' (top tier) the predictions are indicated by orange = upregulated.

Supplementary file 8 : 45 minutes of exposure to 25 μM of PK 11195 of U118MG cells. List of 'Regulators' of figure of file 7, detailing their known modulation of the genes of the 'Data Set' and the consequential 'Effects'.

	**Upstream Regulator**	**Molecule Type**	**Target genes in dataset with changed expression P<0.5**	**Effects**
**A'**	ERK	[RNA-polymerase]-subunit kinase	**CTGF↑, EGR1↑, CXCL8↑, FOS↑, PTGS2 ↑**	a,b,c
**B'**	EGFR	epidermal growth factor receptor	**CTGF↑, EGR1↑, CXCL8↑, FOS↑, PTGS2↑**	a,b,c
**C'**	STAT3	signal transducer and activator of transcription 3	**SGK1↑, EGR1↑, CXCL8↑, FOS↑, PTGS2↑ , NR4A2↑**	a,b,c
**D'**	TNF	tumor necrosis factor	**SGK1↑, EGR1↑, CXCL8↑, FOS↑, PTGS2↑ , ATF3↑, NR4A2↑**	a,b,c
**E'**	CREB1	cAMP responsive element binding protein 1	**EGR1↑, CXCL8↑, CYR61↑, FOS↑, PTGS2↑ , ATF3↑, NR4A2↑**	unknown
**F'**	PDGF BB	Pdgf (complex)	**CTGF↑, SGK1↑, EGR1↑, CXCL8↑, CYR61↑, FOS,↑ PTGS2↑, ATF3↑, NR4A2↑**	a,b,c
**G'**	F2	coagulation factor II, thrombin	**CTGF↑, EGR1↑, CXCL8↑, FOS↑, PTGS2↑**	a,c
**H '**	FOXO1	forkhead box O1	**CTGF↑, SGK1↑, EGR1↑, CXCL8↑, FOS↑**	c
**I'**	FOXO3	forkhead box O3	**CTGF↑, SGK1↑,EGR1↑, CXCL8↑, FOS↑**	unknown

Table Supplementary file 8. Details regarding the 'Regulators' of the figure of File 7. List of primed capital lettering (first column) matches the 'Regulators' marked with the same lettering in the upper tier of Figure 5. The acronyms of the 'Regulators are given (second column). The molecular type of the 'Regulators' is summarized (third column). The gene symbols of the target genes of each of the 'Regulators' are listed (fourth column). Upregulation of gene expression is indicated in **'bold font'** and the corresponding arrows. The final 'Effects' (fifth column) induced by the 'Regulators' and their targeted genes, are indicated with lower case lettering in the fifth column, according to the same lettering in Figure 3 marking the 'Effects' in the lower tier. Thus each Regulator affects a group of genes associated with a group of functions. Furthermore, these groups of genes and functions overlap. This configuration is defined as a super-assembly (see text for more detailed explanations).

Supplementary file 9 : 45 minutes of exposure of U118MG cells to PK 11195 (25 µM).

(canonical pathway as provided by pathway analysis with Ingenuity IPA® showing that also at 45 minutes of exposure to 25 µM of PK 11195 genes are upregulated for components that are part of the canonical pathway for regulation of angiogenesis and tumor growth).

The genes in question are marked in red and light red.

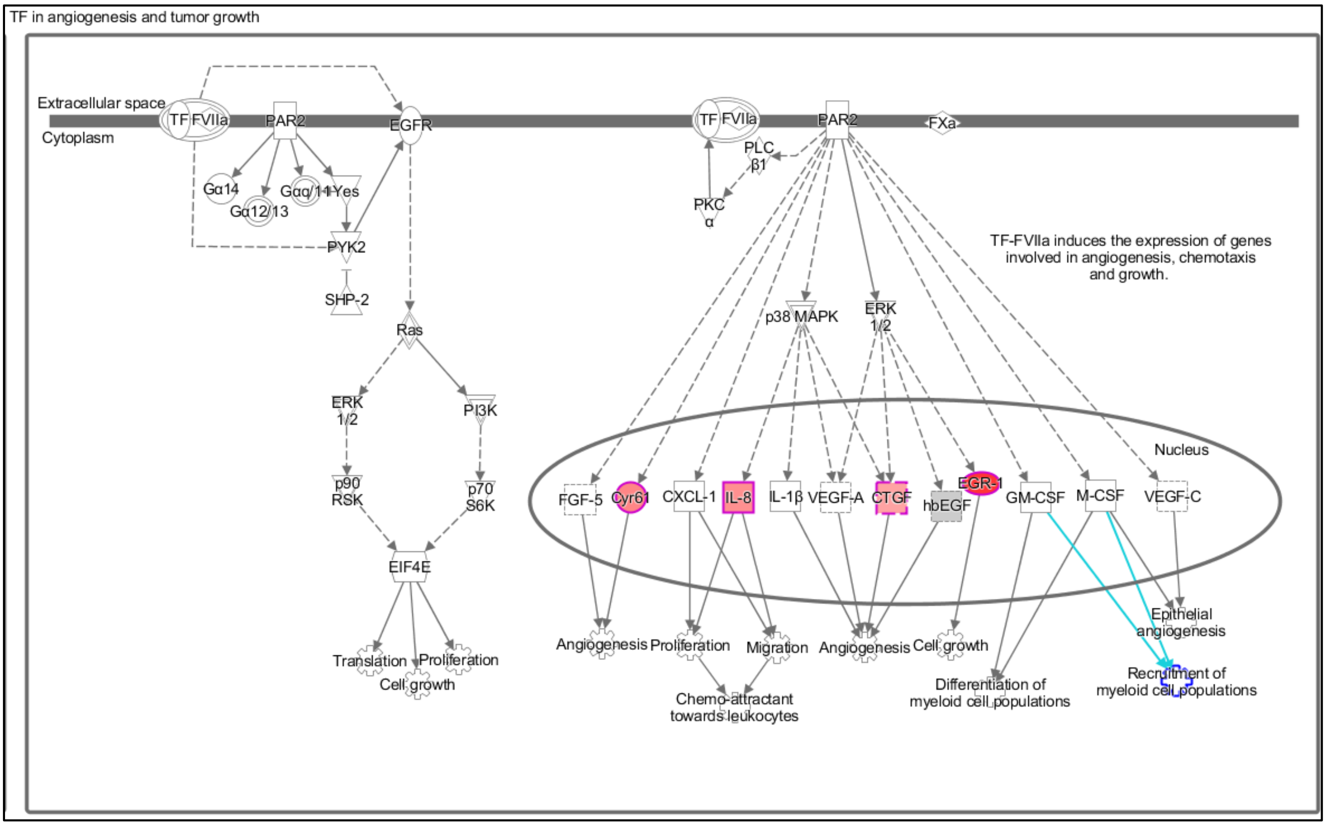

Supplementary file 10 : 1 hour of exposure to 25 µM of PK 11195 of U118MG cells

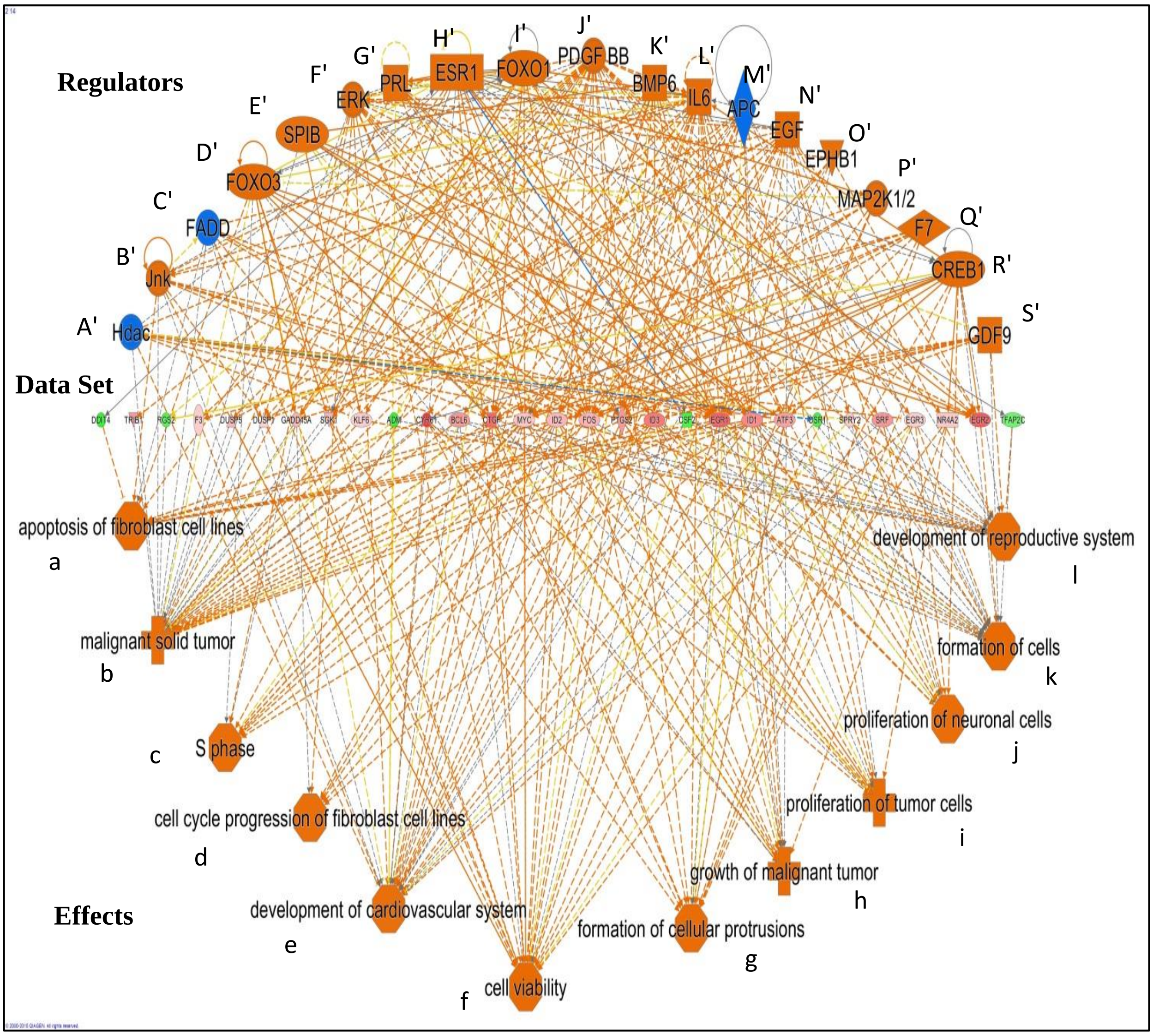

The Genes of the Data Set from left to right: DDIT4↓ TRIB1↑ RGS2↓ F3↑ DUSP5↑ DUSP1↑ GADD45A↑ SGK1↑ KLF6↑ ADM↓ CYR61↑ BCL6↑ CTGF↑ MYC↑ ID2↑ FOS↑ PTGS2↑ ID3↑ CSF2↓ EGR1↑ ID1↑ ATF3↑ OSR1↓ SPRY2↑ SRF↑ EGR3↑ NR4A2↑ EGR2↑ TFAP2C↓

Potential effects on cellular functions due to the significant changes in gene expression as induced by 1 hour of exposure of U118MG cells to PK 11195 (25 µM). The pathway analysis (Regulator Effects analytic IPA® applying adjusted p ≤ 0.05) indicates multiple interactive upstream pathways ('Regulators' given in the upper tier) related to the genes with significantly changed expression ('Data Set' given in the middle tier), together with the various downstream functions ('Effects' in the bottom tier). This whole configuration can be annotated as a super-assembly. Color coding: In the 'Data Set' (middle tier): pink/red = upregulated, green = down regulated. In the 'Effects' (bottom tier) and in the 'Regulators' (top tier) the predictions are indicated by blue = downregulated, and orange = upregulated. The row of gene symbols at the bottom of the figure are those of the middle tier ('Data Set') of this figure presented in the same sequence from left to right. The color coding in the figure indicates upregulation (red) and downregulation (blue or green).

Supplementary file 11 : 1 hour of exposure of U118MG cells to PK 11195 (25 µM).

List of 'Regulators' of figure of file 10 (1 hour of 25 µM of PK 11195), detailing their known modulation of the genes of the 'Data Set' and the consequential 'Effects'.

	Upstream Regulator	Molecule Type	Target genes in dataset with changed expression p < 0.05	Effects
**A'**	Hdac	group (Histone deacetylases-enzymes)	**ATF3↑, BCL6↑, EGR1↑, EGR3↑, FOS↑, KLF6↑, SPRY2↑,** OSR1↓	a,d
**B'**	Jnk	group of MAP kinases	**CTGF↑, DUSP1↑, EGR1↑, FOS↑, PTGS2↑**	a,b,e,f,g
**C'**	FADD	adaptor protein	**EGR1↑, FOS↑, KLF6↑, MYC↑**	a,b,e,f
**D'**	FOXO3	transcription regulator	**CTGF↑, EGR1↑, EGR2↑, FOS↑, FOSB↑, GADD45A↑, SGK1↑**	a,b,c,e,f,h,i
**E'**	SPIB	transcription regulator	**ATF3↑, EGR1↑, EGR2↑, KLF6↑**	b,h,i,k
**F'**	ERK1/2	group (kinases)	**CTGF↑, EGR1↑, F3↑, FOS↑, ID1↑, MYC↑, PTGS2↑, SGK1↑,** CSF2↓	b,c,d,e,f,g,j
**G'**	PRL	cytokine	**EGR1↑, ID1↑, ID3↑, MYC↑**	b,e,i,k,l
**H'**	ESR1	ligand dependent nuclear receptor	**EGR1↑, FOS↑, ID1↑, MYC↑, PTGS2↑, SGK1↑,** CSF2↓, DDIT4↓, TFAP2C↓	a,b,e,f,j,l
**I'**	FOXO1	transcription regulator	**CTGF↑, EGR1↑, EGR2↑, FOS↑, FOSB↑, GADD45A↑, SGK1↑**	b,e,f,j,k
**J'**	PDGF BB	complex (growth factors)	**ATF3↑, CBX4↑, CTGF↑, CYR61↑, DUSP1↑, DUSP5↑, EGR1↑, EGR2↑, EGR3↑, F3↑, FOS↑, FOSB↑, GADD45A↑, KLF6↑, MYC↑, NR4A2↑, PTGS2↑, SGK1↑, SRF↑, TRIB1↑,** ADM↓, CSF2↓, RGS2↓	b,c,e,f,g,j,k
**K'**	BMP6	growth factor	**CTGF↑, ERRFI1↑, ID1↑, ID2↑, ID3↑, PTGS2↑**	b,e,f,h,j,l
**L'**	IL6	cytokine	**BCL6↑, EGR1↑, FOS↑, ID1↑, ID2↑, MYC↑, PTGS2↑, SGK1↑**	b,c,e,f,h,i,j
**M'**	APC	enzyme	**ID1↑, ID2↑, ID3↑, MYC↑, PTGS2↑, SGK1↑**	b,c,e,f,h,i,k,
**N'**	EGF	growth factor	**DUSP1↑, DUSP5↑, EGR1↑, EGR2↑, FOS↑, MYC↑, PTGS2↑, SPRY2↑**	b,c,f,g,h,i,k,l
**O'**	EPHB1	kinase	**EGR1↑, EGR2↑, FOS↑, PTGS2↑**	e
**P'**	MAP2K1/2	group (kinases)	**ATF3↑, CTGF↑, DUSP1↑, EGR1↑, MYC↑**	unknown
**Q'**	F7	peptidase	**CTGF↑, EGR1↑, F3↑, FOS↑, GADD45A↑, MYC↑**	f,g
**R'**	CREB1	transcription regulator	**ATF3↑, CYR61↑, EGR1↑, EGR2↑, ERRFI1↑, FOS↑, FOSB↑, MYC↑, NR4A2↑, PTGS2↑,** RGS2↓	f,g,k,l
**S'**	GDF9	growth factor	**CTGF↑, ID1↑, ID2↑, ID3↑, PTGS2↑**	k,l

Table Supplementary file 11. Details regarding the 'Regulators' of the figure of file 10. This table lists details regarding the 'Regulators' for 1 hour of PK 11195 exposure. The list of primed capital lettering (first column) matches the 'Regulator**s'** marked with the same lettering in the upper tier of the figure of supplementary file 10. The acronyms of the 'Regulators are given (second column). The molecular type of the 'Regulators' is summarized (third column). The target genes of each of the 'Regulators' are listed (fourth column). Upregulation of gene expression is indicated in **'bold font'** and the corresponding arrows. Downregulation is indicated in 'grey font' and downward pointing arrows. The final 'Effects' (fifth column) induced by the 'Regulators' and their targeted genes, are indicated with lower case lettering in the fifth column, according to the same lettering in the figure of supplementare file 10 marking the 'Effects' in the lower tier. Thus each Regulator affects a group of genes associated with a group of functions. Furthermore, these groups of genes and functions overlap. This configuration is defined as a super-assembly (see text for more detailed explanations).

Supplementary file 12 : 3 hours of exposure to 25 µM of PK 11195 of U118MG cells

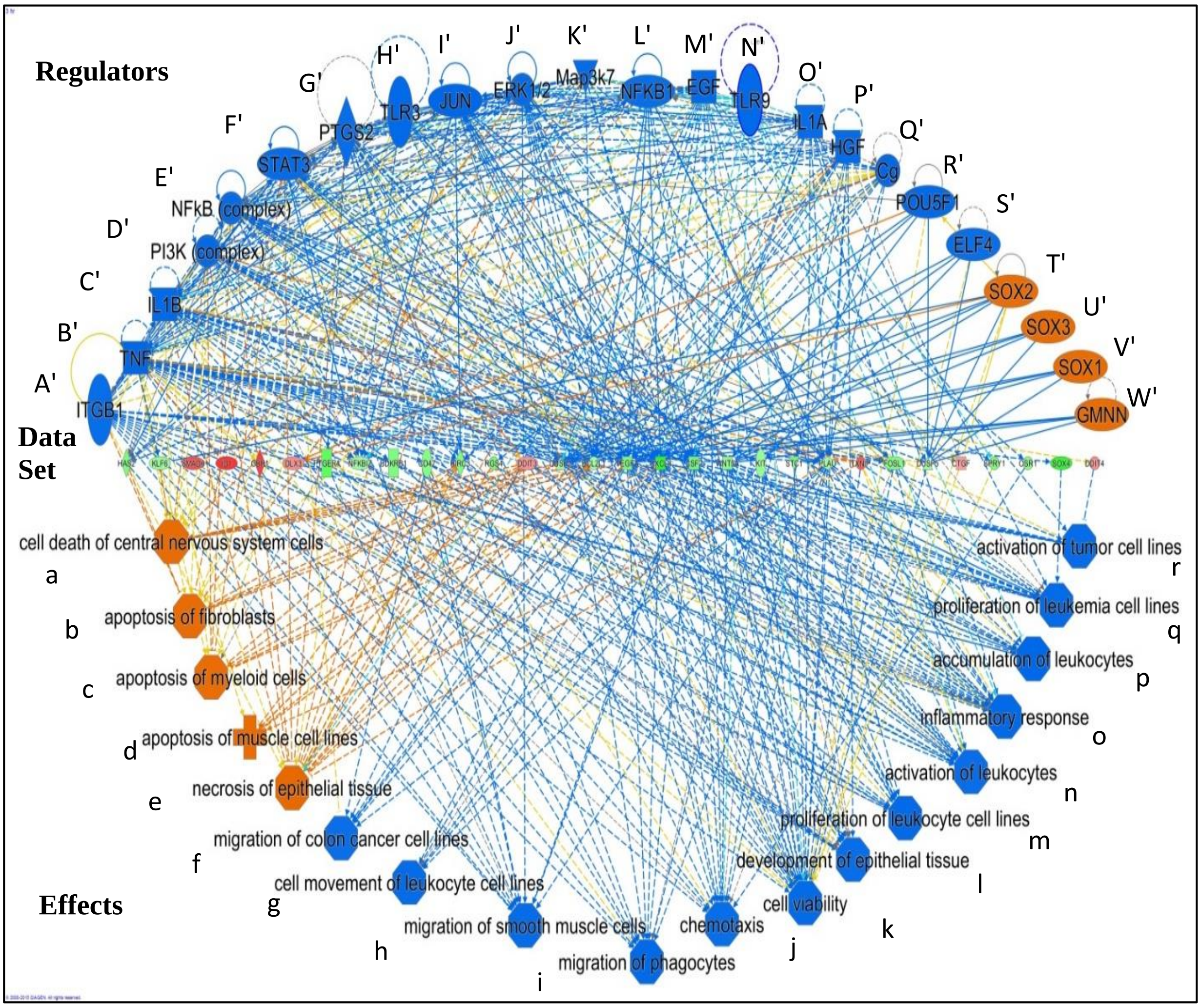

The genes of the Data Set from left to right: HAS2↓, KLF6↓, SMAD6↑ ID1↑ GBP1↑ DLX3↑ PTGER4↓ NFKBIZ↓ BDKRB1↓ CD47↓ BIRC3↓ RGS4↓ DDIT3↑ DUSP1↓ BCL2L1↓ VEGFA↓ CXCL8↓ CSF2↓ WNT5A↓ KIT↓ STC1↓ PLAU↓ TXNIP↑ FOSL1↓ DUSP5↓ CTGF↑ SPRY1↓ OSR1↓ SOX4↓ DDIT4↑

Potential effects on cellular functions due to the significant changes in gene expression as induced by 3 hours of exposure of U118MG cells to PK 11195 (25 µM). The pathway analysis (Regulator Effects analytic IPA® applying adjusted p ≤ 0.05) indicates multiple interactive upstream pathways ('Regulators' given in the upper tier) related to the genes with significantly changed expression ('Data Set' given in the middle tier), together with the various downstream functions ('Effects' in the bottom tier). This whole configuration can be annotated as a super-assembly (see text for detailed explanation). Color coding: In the 'Data Set' (middle tier): pink/red = upregulated, green = down regulated. In the 'Effects' (bottom tier) and in the 'Regulators' (top tier) the predictions are indicated by blue = downregulated, and orange = upregulated. The row of gene symbols at the bottom of the figure are those of the middle tier ('Data Set') of this figure presented in the same sequence from left to right. The color coding indicates upregulation (red) and downregulation (green or blue).

Supplementary file 13 : 3 hours of exposure of U118MG cells to PK 11195 (25 µM).

List of 'Regulators' of the figure of file 12 (3 hours of 25 µM of PK 11195), detailing their known modulation of the genes of the 'Data Set' and the consequential 'Effects' .

	Upstream Regulator	Molecule Type	Target genes in dataset with changed expression P<0.05	Effects
A'	ITGB1	transmembrane receptor	CXCL8↓, PLAU↓, PTGER4↓, VEGFA↓, WNT5A↓	e,h,i,j,k,l,m,n,o
B'	TNF	cytokine	**ATF4↑, DLX1↑, GBP1↑, ID1↑, TAGLN↑, TXNIP↑,** BCL2L1↓, BDKRB1↓, BIRC3↓, CD47↓, CSF2↓, CXCL8↓, DUSP1↓, DUSP5↓, FOSL1↓, KLF6↓, NFKBIZ↓, PHLDA1↓, PLAU↓, RGS2↓, SOX4↓, VEGFA↓, WNT5A↓	b,c,d,e,h,i,j,k,l,n,o, p,m,q,r
C'	IL1B	cytokine	**ATF4↑, DDIT4↑, FOSB↑ ,TXNIP↑,** BDKRB1↓, BIRC3↓, CSF2↓, CXCL8↓, DUSP1↓, FOSL1↓, HAS2↓, NFKBIZ↓, PHLDA1↓, PLAU↓, VEGFA↓	a,b,c,e,h,i,j,k,l,m, n,o,p,q
D'	PI3K	complex (kinase)	**DDIT3↑, TXNIP↑,** BCL2L1↓, CXCL8↓, DUSP1↓, FOSL1↓, RGS2↓, VEGFA↓	a,b,c,e,h,i,j,k,o,p, m,q
E'	NFKB	Complex (transcription regulator)	**CTGF↑, DDIT3↑,** BCL2L1↓, BIRC3↓, CSF2↓, CXCL8↓, DUSP5↓, HAS2↓, KIT↓, NFKBIZ↓, PLAU↓	c,e,i,k,l,n,o,p,q,r
F'	STAT3	transcription regulator	**DDIT3↑, GLIPR1↑,** BCL2L1↓, CSF2↓, CXCL8↓, HAS2↓, NFKBIZ↓, PHLDA1↓, VEGFA↓	b,c,e,f,g,h,k,m,n,o, p,q,r
G'	PTGS2	enzyme	BCL2L1↓, BIRC3↓, CXCL8↓, DUSP1↓, PTGER4↓, ST3GAL1↓, VEGFA↓	a,e,k,l,n,o,h,i,j
H'	TLR3	transmembrane receptor	CSF2↓, CXCL8↓, DUSP1↓, NFKBIZ↓, PHLDA1↓, SPRY1↓	b,c,e,k,n,o,p
I'	JUN	transcription regulator	BIRC3↓, CSF2↓, CXCL8↓, DUSP1↓, FOSL1↓, NFKBIZ↓, PLAU↓, VEGFA↓, WNT5A↓	a,b,e,j,k,l,m
J'	ERK1/2	Group (kinase)	**CTGF↑, ID1↑,** BCL2L1↓, CSF2↓, CXCL8↓, FOSL1↓, HAS2↓, PLAU↓, VEGFA↓	a,c,e,h,i,j,k,l,o,m
K'	Map3k7	kinase	**OSR1↑,** BCL2L1↓, CSF2↓, HAS2↓, NFKBIZ↓	b,e,k,o,p
L'	NFKB1	transcription regulator	BCL2L1↓, CSF2↓, CXCL8↓, HAS2↓, PLAU↓, VEGFA↓	a,e,k,n,o,q
M'	EGF	growth factor	BCL2L1↓, CXCL8↓, DUSP1↓, DUSP5↓, ST3GAL1↓, VEGFA↓	b,e,f,j,k,l,m,r
N'	TLR9	transmembrane receptor	CSF2↓, CXCL8↓, DUSP1↓, NFKBIZ↓, PHLDA1↓, SPRY1↓	e,j,k,n,o,p
O'	IL1A	cytokine	BIRC3↓, CSF2↓, CXCL8↓, NFKBIZ↓, PLAU↓	b,f,h,k,n,o,q
P'	HGF	growth factor	**CTGF↑,** BCL2L1↓, BIRC3↓, CXCL8↓, PLAU↓, VEGFA↓	b,d,e,f,i,j,k,l
Q'	Cg	complex	**SMAD6↑,** BCL2L1↓, CCNE2↓, CXCL8↓, DUSP1↓, HAS2↓, PHLDA1↓, PLAU↓, RGS2↓, RGS4↓, STC1↓, VEGFA↓	unknown
R'	POU5F1	transcription regulator	**DLX1↑, DLX3↑,** DUSP1↓, DUSP5↓, IER5↓, VEGFA↓, WNT5A↓	k
S'	ELF4	transcription regulator	CXCL8↓, DUSP1↓, DUSP5↓, KIT↓	k
T'	SOX2	transcription regulator	**CTGF↑, DLX1↑, DLX3↑,** DUSP1↓, DUSP5↓, IER5↓, KLF6↓, VEGFA↓, WNT5A↓	k
U'	SOX3	transcription regulator	DUSP1↓, DUSP5↓, IER5↓, VEGFA↓, WNT5A↓	unknown
V'	SOX1	transcription regulator	DUSP1↓, DUSP5↓, IER5↓, VEGFA↓, WNT5A↓	unknown
W'	GMNN	transcription regulator	DUSP1↓, DUSP5↓, IER5↓, VEGFA↓, WNT5A↓	unknown

Details regarding the 'Regulators' of **supplementary file 13**. The presentation is organized in the same way as in the Table of supplementary file 11.

Supplementary file 14 (Figure 4 A – E in the ms.) tumorigenicity 'Effects' associated with changes in gene expression due to exposure of U118MG cells to 25 μM of PK 11195.

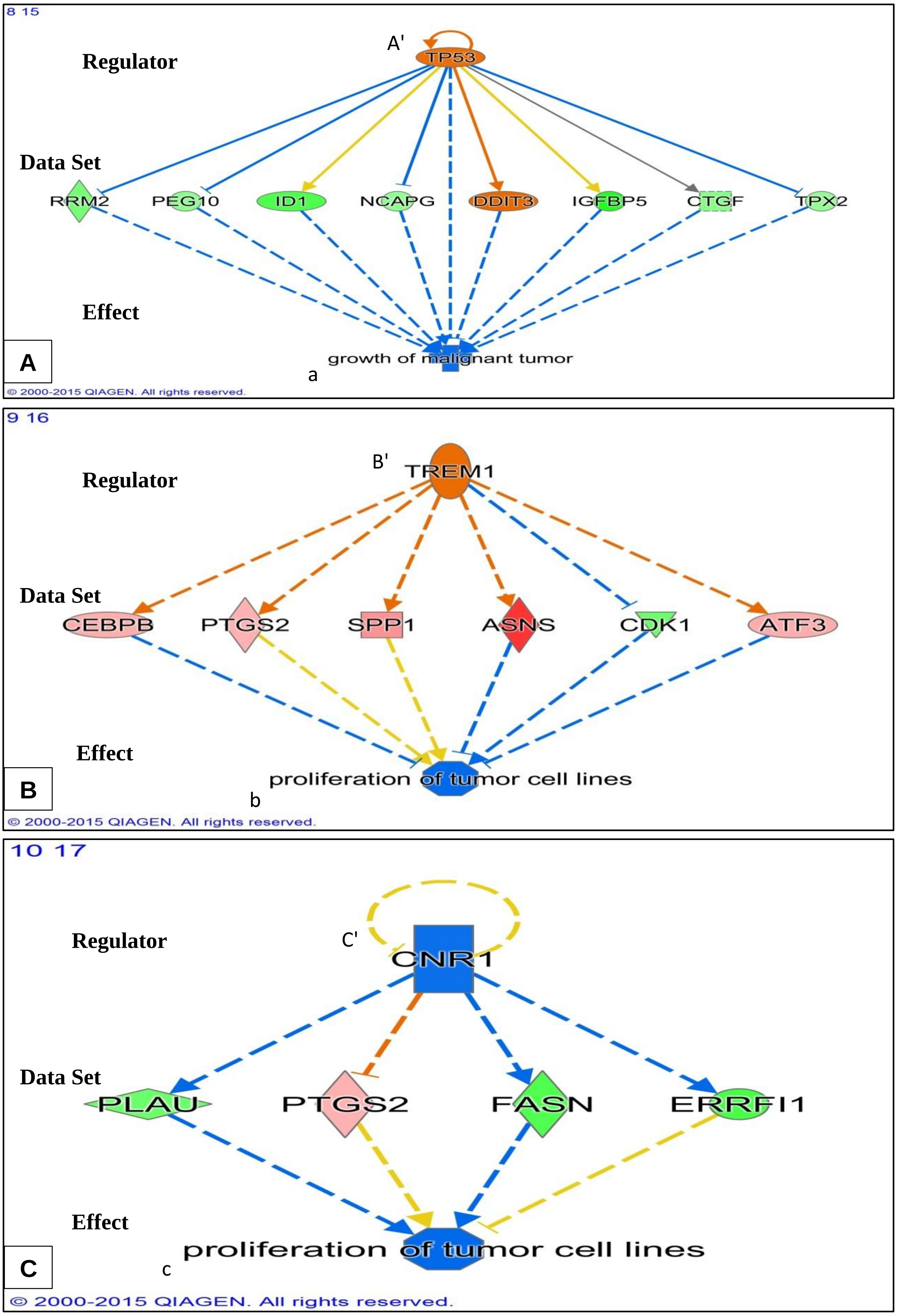

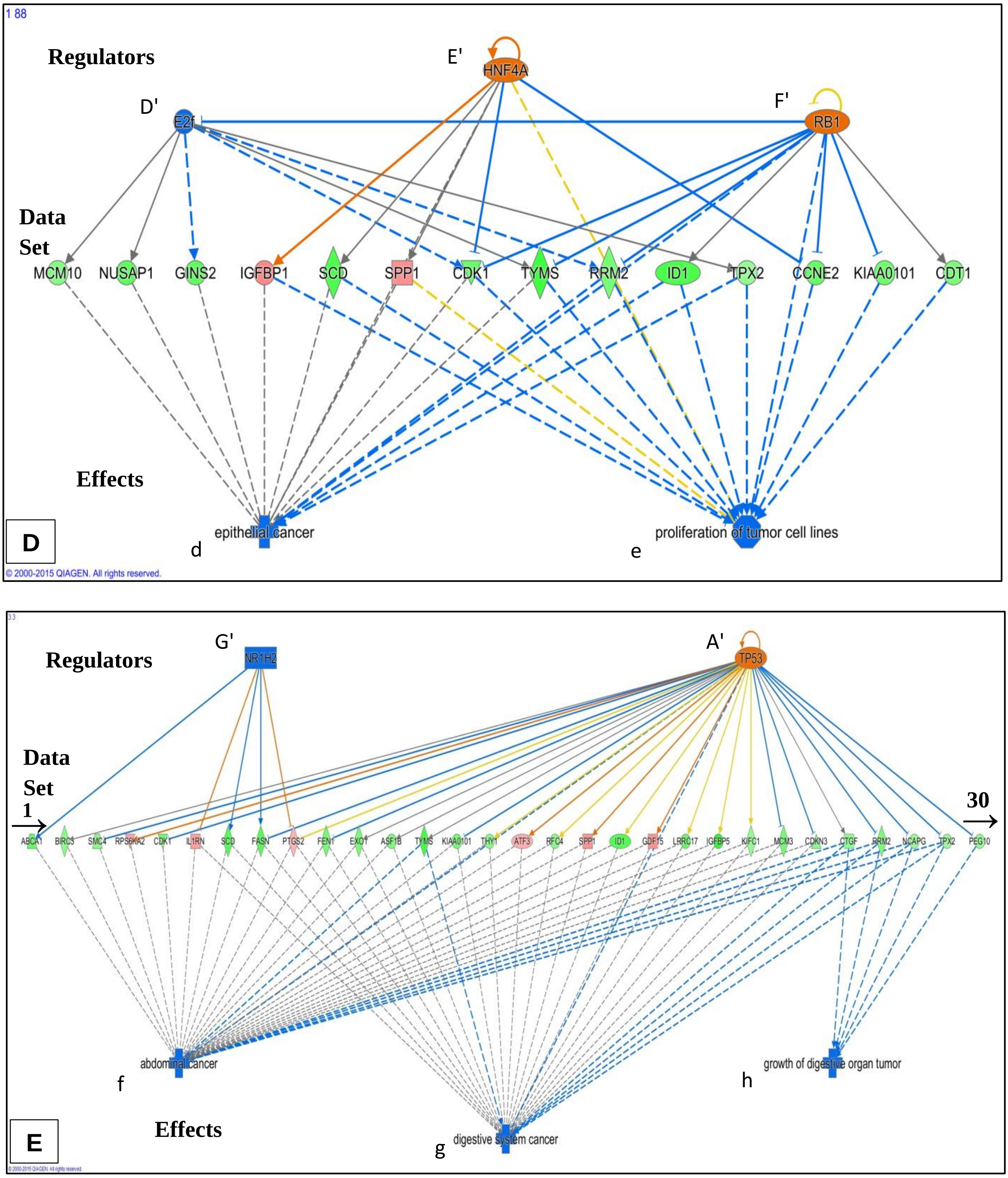

The genes of the Data Set in 'E' from left to right: ABCA1↓ BIRC3↓ SMC4↓ RPS6KA2↑ CDK1↓ IL1RN↑ SCD↓ FASN↓ PTGS2↑ FEN1↓ EXO1↓ ASF1B↓ TYMS↓ KIAA0101↓ THY1↓ ATF3↑ RFC4↓ SPP1↑ ID1↓ GDF15↑ LRRC17↓ IGFBP5↓ KIFC1↓ MCM3↓ CDKN3↓ CTGF↓ RRM2↓ NCAPG↓ PEG10

Potential effects on tumorigenicity due to gene expression following 24 hrs of exposure of U118MG cells to PK 11195 (25 μM). This is Figure 4A-E in the manuscript. As found with Regulator Effects analyticIPA® applying adjusted p ≤ 0.05, in **A,B,C,** individual 'Regulators' (given in the upper tiers) are related to specific groups of genes with significantly changed expression ('Data Sets' given in the middle tiers), together with their particular downstream functions ('Effects' in the bottom tiers), namely, suppression of growth of malignant tumor (in A) and suppression of proliferation of tumor cell lines (in B and C). In **D,** three 'Regulators' are upstream to an array of genes (the 'Data Set' given in the middle tier), with significantly changed expression. This 'Data Set' is subdivided into two subsets that exert two particular downstream functions ('Effects' in the bottom tier), namely suppression of epithelial cancer and suppression of proliferation of tumor cells. In **E**, two 'Regulators' are upstream to an array of

genes (the 'Data Set' given in the middle tier) with significantly changed expression. This 'Data Set' is subdivided into three subsets that exert three particular downstream functions ('Effects' in the bottom tier), namely suppression of abdominal cancer, suppression of digestive system cancer, and suppression of growth of digestive organ tumor. Each mentioned separate set can be considered an assembly of pathways running from 1 or few Regulators via a number of genes to affect not more than 1 or 2 specific functions. Color coding: pink/orange = upregulated, blue/green = down regulated. The configurations in seen in A – C can be considered assemblies. The configurations in seen in D – E can be considered super-assemblies (for more detailed explaination see the text).

Supplementary file 15 : List of 'Regulators' of supplementary file 14 (24 hours of 25 µM of PK 11195), detailing their known modulation of the genes of the 'Data Sets' and the consequential 'Effects' (tumorigenicity).

	Upstream Regulator	Molecule Type	Target genes in dataset with changed expression P<0.05	Effects
**A'**	TP53	transcription regulator	**ATF3↑, DDIT3↑, GDF15↑, PTGS2↑, RPS6KA2↑, SPP1↑,** ASF1B↓, BIRC3↓, CDK1↓, CDKN3↓, CTGF↓, EXO1↓, FASN↓, FEN1↓, ID1↓, IGFBP5↓, KIAA0101↓, KIFC1↓, LRRC17↓, MCM3↓, NCAPG↓, PEG10↓, RFC4↓, RRM2↓, SMC4↓, THY1↓, TPX2↓, TYMS↓	a,f,g,h
**B'**	TREM1	transmembrane receptor	**ASNS↑, ATF3↑, CEBPB↑, PTGS2↑, SPP1↑,** CDK1↓	b
**C'**	CNR1	g-protein coupled receptor	**PTGS2↑,** ERRFI1↓, FASN↓, PLAU↓	c
**D'**	E2f	Group (transcription regulator)	CDK1↓, GINS2↓, MCM10↓, NUSAP1↓, RRM2↓, TPX2↓, TYMS↓	unknown
**E'**	HNF4A	transcription regulator	**IGFBP1↑, SPP1↑,** CCNE2↓, CDK1↓, SCD↓	d,e
**F'**	RB1	transcription regulator	CCNE2↓, CDK1↓, CDT1↓, ID1↓, KIAA0101↓, RRM2↓, TYMS↓	d,e
**G'**	NR1H2	ligand-dependent nuclear receptor	**IL1RN↑, PTGS2↑,** ABCA1↓, FASN↓, SCD↓	f,g

Details regarding the 'Regulators' of **Figure 4 in the text** and supplementary file 14 that are related to tumorigenicity. The list of primed capital lettering (first column) matches the 'Regulator**s'** marked with the same lettering in the upper tiers of Figures 4A,B,C,D,E and supplementary file 14. The presentation is organized in the same way as in the Table of supplementary files 11 and 13.

Supplementary file 16 (Figure 5 A – C in the ms.) tumorigenicity 'Effects' associated with changes in gene expression due to 24 hours of exposure of U118MG cells to 25 µM of PK 11195.

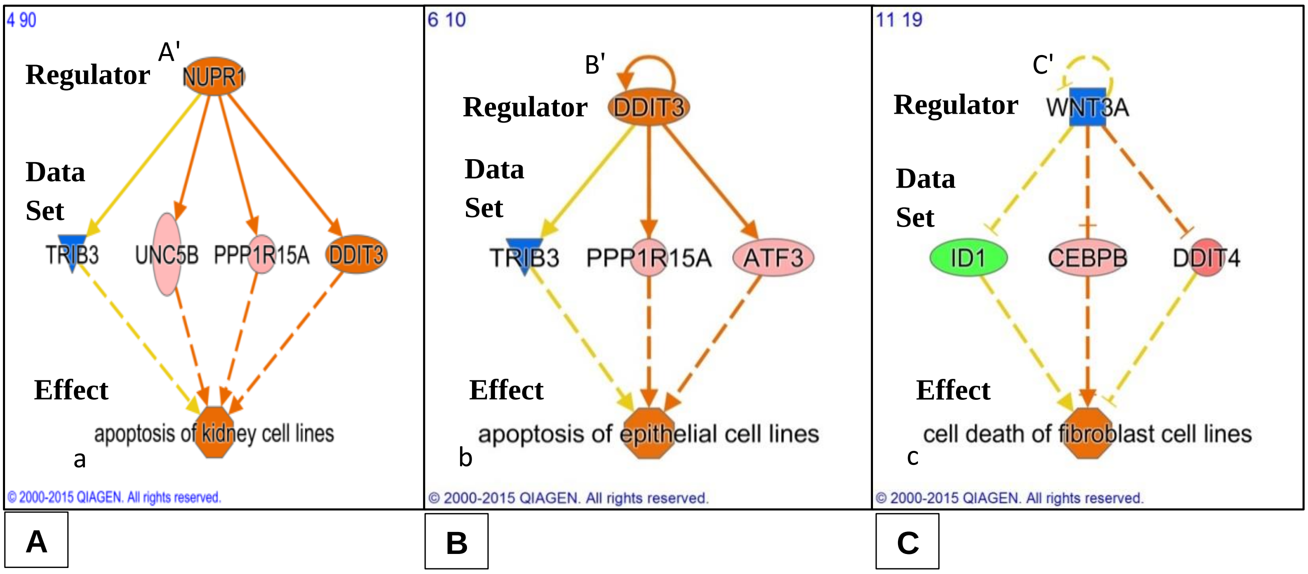

Potential effects on programmed cell death due to gene expression following 24 hrs of exposure of U118MG cells to PK 11195 (25 µM). As analyzed with Regulator Effects analytic; IPA®; applying adjusted $p \leq 0.05$), in A,B,C, individual 'Regulators' (given in the upper tiers) are related to specific groups of genes with significantly changed expression ('Data Sets' given in the middle tiers), together with their particular downstream functions ('Effects' in the bottom tiers), namely, stimulation of apoptosis of kidney cell lines (in A), stimulation of apoptosis of epithelial cell lines (in B), stimulation of cell death of fibroblast cell lines (in C). Each mentioned separate set can be considered an assembly of pathways running from 1 Regulator via a number of genes to affect not more than 1 specific function. Color coding: pink/orange = upregulated, blue/green = down regulated. For more detailed explanation see text.

Supplementary file 17 List of 'Regulators' of (figure 5 in the text, 24 hours of 25 µM of PK 11195), detailing their known modulation of the genes of the 'Data Sets' and the consequential 'Effects' (programmed cell death).

	Upstream Regulator	Molecule Type	Target genes in dataset with changed expression P<0.05	Effects
**A'**	NUPR1	transcription regulator	**DDIT3↑, PPP1R15A↑, TRIB3↑, UNC5B↑**	a
**B'**	DDIT3	transcription regulator	**ATF3↑, DDIT3↑, PPP1R15A↑ , TRIB3↑**	b
**C'**	WNT3A	cytokine	**CEBPB↑, DDIT4↑,** ID1↓	c

Details regarding the 'Regulators' of the figure of supplementary file 16 **(Figure 5 in text)** that are related to programmed cell death. The list of primed capital lettering (first column) matches the 'Regulator**s'** marked with the same lettering in the upper tiers of of supplementary file 16 (Figures 5A,B,C in the text). The presentation is organized in the same way as in the Table of supplementary file 11.

Supplementary file 18 List of gene symbols for genes with changed expression due to PK 11195 exposure in the present study.

abbreviation	Full name	abbreviations	Full name	abbreviations	Full name
**ABCA1**	ATP-binding cassette, sub-family A (ABC1), member 1	**FOSB**	FBJ murine osteosarcoma viral oncogene homolog B	**PLEKHF1**	pleckstrin homology domain containing, family F (with FYVE domain) member 1
**ACTG2**	actin, gamma 2, smooth muscle, enteric	**FOSL1**	FOS-like antigen 1	**PPP1R15A**	protein phosphatase 1, regulatory subunit 15A
**ADM**	adrenomedullin	**GADD45A**	growth arrest and DNA-damage-inducible, alpha	**PSAT1**	phosphoserine aminotransferase 1
**ANP32AP1**	acidic nuclear phosphoprotein 32 family member A pseudogene 1	**GBP1**	guanylate binding protein 1, interferon-inducible	**PTGER4**	prostaglandin E receptor 4
**ASF1B**	anti-silencing function 1B histone chaperone	**GDF15**	growth differentiation factor 15	**PTGS2**	prostaglandin-endoperoxide synthase 2
**ASNS**	asparagine synthetase (glutamine-hydrolyzing)	**GINS2**	GINS complex subunit 2 (Psf2 homolog)	**PTMA**	prothymosin, alpha
**ATF3**	activating transcription factor 3	**HAS2**	hyaluronan synthase 2	**RFC4**	replication factor C (activator 1) 4, 37kDa
**ATOH8**	atonal bHLH transcription factor 8	**HBEGF**	heparin binding EGF like growth factor	**RGS2**	regulator of G-protein signaling 2
**BCL2CL1**	BCL2-like 1	**HMGB2**	high mobility group box 2	**RGS4**	regulator of G-protein signaling 4
**BCL6**	B-cell CLL/lymphoma 6	**ID1**	inhibitor of DNA binding 1,	**RNA28S5**	RNA, 28S ribosomal 5
**BDKRB1**	bradykinin receptor B1	**ID2**	inhibitor of DNA binding 2	**RPS6KA2**	ribosomal protein S6 kinase, 90kDa, polypeptide 2
**BEX2**	brain expressed X-linked 2	**ID3**	inhibitor of DNA binding 3	**RPL21P28**	ribosomal protein L21 pseudogene 28
**BIRC3**	baculoviral IAP repeat containing 3	**IGFBP1**	insulin-like growth factor binding protein 1	**RPS2P28**	ribosomal protein S2 pseudogene 28
**CCNE2**	cyclin E2	**IGFBP5**	insulin-like growth factor binding protein 5	**RRM2**	ribonucleotide reductase M2
**CD47**	CD47 molecule	**IL1RN**	interleukin 1 receptor antagonist	**SCD**	stearoyl-CoA desaturase (delta-9-desaturase)
**CDK1**	cyclin-dependent kinase 1	**IL8**	Interleukin 8	**SGK**	serum/glucocorticoid regulated kinase
**CDKN3**	cyclin-dependent kinase inhibitor 3	**IL18**	interleukin 18	**SGK1**	serum/glucocorticoid regulated kinase 1
**CDT1**	chromatin licensing and DNA replication factor 1	**KIAA0101**	KIAA0101	**SLC1A5**	solute carrier family 1 (neutral amino acid transporter), member 5
**CEBPB**	CCAAT/enhancer binding protein (C/EBP), beta	**KDM3A**	lysine demethylase 3A	**SLC3A2**	solute carrier family 3 (amino acid transporter heavy chain), member 2
**CLK1**	CDC like kinase 1	**KIFC1**	kinesin family member C1	**SLC6A15**	solute carrier family 6 (neutral amino acid transporter), member 15
**CSF2**	colony stimulating factor 2	**KIT**	v-kit Hardy-Zuckerman 4 feline sarcoma viral oncogene homolog	**SLC7A5**	solute carrier family 7 (amino acid transporter light chain, L system), member 5
**CTGF**	connective tissue growth factor	**KLF6**	Kruppel-like factor 6	**SMAD6**	SMAD family member 6
**CXCL8**	chemokine (C-X-C motif) ligand 8	**LOC100507 412**	uncharacterized LOC100507412	**SMC4**	structural maintenance of chromosomes 4
**CYR61**	cysteine-rich, angiogenic inducer, 61	**LOC729779**	phosphoserine aminotransferase 1 pseudogene 3	**SOX4**	SRY (sex determining region Y)-box 4
**DDIT3**	DNA-damage-inducible transcript 3	**LRRC17**	leucine rich repeat containing 17	**SPRR2D**	small proline-rich protein 2D
**DDIT4**	DNA-damage-inducible transcript 4	**MCM3**	minichromosome maintenance complex component 3	**SPP1**	secreted phosphoprotein 1
**DLX3**	distal-less homeobox 3	**MCM10**	minichromosome maintenance 10 replication initiation factor	**SPRY1**	sprouty RTK signaling antagonist 1
**DMC1**	DNA meiotic recombinase 1	**MIR22HG**	MIR22 host gene	**SPRY2**	sprouty RTK signaling antagonist 2
**DUSP1**	dual specificity phosphatase 1	**MYC**	v-myc avian myelocytomatosis viral oncogene homolog	**SRF**	serum response factor
**DUSP5**	dual specificity phosphatase 5	**MYLIP**	myosin regulatory light chain interacting protein	**STC1**	stanniocalcin 1
**DYNC1H1**	dynein cytoplasmic 1 heavy chain 1	**NABP**	nucleic acid binding protein 1	**TFAP2C**	transcription factor AP-2 gamma (activating enhancer binding protein 2 gamma)
**EGR1**	early growth response 1	**NCAPG**	non-SMC condensin I complex, subunit G	**TGIF1**	TGFB-induced factor homeobox 1
**EGR2**	early growth response 2	**NEXN**	nexilin (F actin binding protein)	**THY1**	Thy-1 cell surface antigen
**EGR3**	early growth response 3	**NFKBIZ**	nuclear factor of kappa light polypeptide gene enhancer in B-cells inhibitor, zeta	**TPX2**	TPX2, microtubule-associated
**ERRFI1**	ERBB receptor feedback inhibitor 1	**NR4A2**	nuclear receptor subfamily 4, group A, member 2	**TRIB1**	tribbles pseudokinase 1
**EXO1**	exonuclease 1	**NUPR1**	nuclear protein, transcriptional regulator, 1	**TRIB3**	tribbles pseudokinase 3
**F3**	coagulation factor III (thromboplastin, tissue factor)	**NUSAP1**	nucleolar and spindle associated protein 1	**TUFT1**	tuftelin 1
**FAM102A**	family with sequence similarity 102, member A	**OSR1**	odd-skipped related transciption factor 1	**TXNIP**	thioredoxin interacting protein
**FASN**	fatty acid synthase	**P8**	nuclear protein, transcriptional regulator, 1(also known as NUPR1)	**TYMS**	thymidylate synthetase
**FEN1**	flap structure-specific endonuclease 1	**PCK2**	phosphoenolpyruvate carboxykinase 2 (mitochondrial)	**UHRF1**	ubiquitin-like with PHD and ring finger domains 1
**FHL2**	four and a half LIM domains 2	**PCNP**	PEST proteolytic signal containing nuclear protein	**UNC5B**	unc-5 netrin receptor B
**FILIP1L**	filamin A interacting protein 1-like	**PDE5A**	phosphodiesterase 5A, cGMP-specific	**VEGFA**	vascular endothelial growth factor A
**FKBP10**	FK506 binding protein 10	**PEG10**	paternally expressed 10	**WNK**	lysine deficient protein kinase 1
**FOLR3**	folate receptor 3 (gamma)	**PHGDH**	phosphoglycerate dehydrogenase	**WNT5A**	wingless-type MMTV integration site family, member 5A
**FOS**	FBJ murine osteosarcoma viral oncogene homolog	**PLAU**	plasminogen activator, urokinase		

Supplementary file 19 : List of acronyms of 'Regulators' related to changes in gene expression due to PK 11195 exposure in the present study

abbreviation	Full name	abbreviation	Full name	abbreviation	Full name
**APC**	adenomatous polyposis coli	**GDF9**	growth differentiation factor 9	**PDGF BB**	platelet-derived growth factor beta polypeptide
**BCR (complex)**	B Cell Receptor	**GMNN**	geminin, DNA replication inhibitor	**PI3K**	phosphatidylinositol-4,5-bisphosphate 3-kinase, catalytic subunit alpha
**BMP6**	bone morphogenetic protein 6	**Hdac**	histone deacetylase	**Pka**	cAMP-dependent protein kinase
**Cg**	cathepsin G	**HGF**	hepatocyte growth factor	**POU5F1**	POU class 5 homeobox 1
**CNR1**	cannabinoid receptor 1 (brain)	**IL1B**	interleukin 1 beta	**PRL**	prolactin
**CREB1**	cAMP responsive element binding protein 1p	**HNF4A**	hepatocyte nuclear factor 4, alpha	**PTGS2**	prostaglandin-endoperoxide synthase 2 (prostaglandin G/H synthase and cyclooxygenase)
**CREM**	cAMP responsive element modulator	**IL1A**	interleukin 1, alpha	**RB1**	retinoblastoma 1
**DDIT3**	DNA-damage-inducible transcript 3	**IL1B**	interleukin 1, beta	**SOX1**	SRY (sex determining region Y)-box 1
**EGF**	epidermal growth factor	**IL6**	interleukin 6	**SOX2**	SRY (sex determining region Y)-box 2
EGFR	epidermal growth factor receptor	**ITGB1**	integrin, beta 1	**SOX3**	SRY (sex determining region Y)-box 3
**ELF4**	E74-like factor 4 (ets domain transcription factor)	**Jnk**	c-Jun N-terminal kinase	**SPIB**	Spi-B transcription factor
**ERK**	Protein kinase	**JUN**	jun proto-oncogene	**STAT3**	signal transducer and activator of transcription 3 (acute-phase response factor)
**EPHB1**	EPH receptor B1	**MAP2K1/2**	mitogen-activated protein kinase kinase 1/2	**TICAM1**	toll like receptor adaptor molecule 1
ERBB2	erb-b2 receptor tyrosine kinase 2	**Map3k7**	mitogen-activated protein kinase kinase kinase 7	**TGFB1**	transforming growth factor beta 1
**ERK1/2**	mitogen-activated protein kinase	**Mek**	Mitogen-activated protein kinase kinase	**TLR3**	toll-like receptor 3
**ESR1**	estrogen receptor	**MET**	MET proto-oncogene, receptor tyrosine kinase	**TLR4**	toll like receptor 4 Synonyms
**F2**	coagulation factor II, thrombin	**MYD88**	myeloid differentiation primary response 88	**TLR9**	toll-like receptor 9
**F7**	coagulation factor VII	**NFKB**	nuclear factor of kappa light polypeptide gene enhancer in B-cells 2 (p49/p100)	**TNF**	tumor necrosis factor
**FADD**	Fas (TNFRSF6)-associated via death domain	**NFKB1**	nuclear factor of kappa light polypeptide gene enhancer in B-cells 1	**TP53**	tumor protein p53
**FOXO1**	forkhead box O1	**NR1H2**	nuclear receptor subfamily 1, group H, member 2	**TREM1**	triggering receptor expressed on myeloid cells 1
**FOXO3**	forkhead box O3	**NUPR1**	nuclear protein, transcriptional regulator, 1	**WNT3A**	wingless-type MMTV integration site family, member 3A
